# On the origins of the sabre-toothed felid model: functional anatomy of the hindlimb in *Promegantereon ogygia* (Felidae, Machairodontinae, Smilodontini) from the Late Miocene of Batallones-1 (Madrid, Spain)

**DOI:** 10.1186/s40850-026-00259-1

**Published:** 2026-03-06

**Authors:** Manuel J. Salesa, Gema Siliceo, Mauricio Antón, Juan F. Pastor

**Affiliations:** 1 Departamento de Paleobiología, Museo Nacional de Ciencias Naturales- CSIC, Madrid, Spain; 2 Fundación ARAID – Agencia Aragonesa para la Investigación y el Desarrollo, Zaragoza, Spain; 3 Fundación Conjunto Paleontológico de Teruel-DINOPOLIS, Teruel, Spain; 4 Departamento de Anatomía, Facultad de Medicina, Universidad de Valladolid, Valladolid, Spain

**Keywords:** Biomechanics, Miocene, Morphology

## Abstract

The Spanish Late Miocene locality of Batallones-1 (Madrid, Spain) has yielded a very rich sample of large carnivorans, including hyaenids, mustelids, amphicyonids, ailurids, felines, and two species of sabre-toothed felids that represent the first radiation event of the subfamily Machairodontinae: *Machairodus aphanistus* and *Promegantereon ogygia*. The former was the size of a tiger and the top predator of the Batallones-1 community, whereas the latter was a much smaller animal, its body weight being similar to that of an extant puma. In the present paper, we study the functional anatomy of the hindlimb of *P. ogygia* compared to that of extant felines and pantherines of similar size. Our observations reveal that the hindlimb of this early sabre-toothed felid, unlike the relatively derived forelimb, show several primitive features also observed in the earliest felid, *Proailurus lemanensis*, thus highlighting the mosaic evolution of machairodontine morphology in this Late Miocene sabre-toothed felid.

## Introduction

**Fig. 1:**
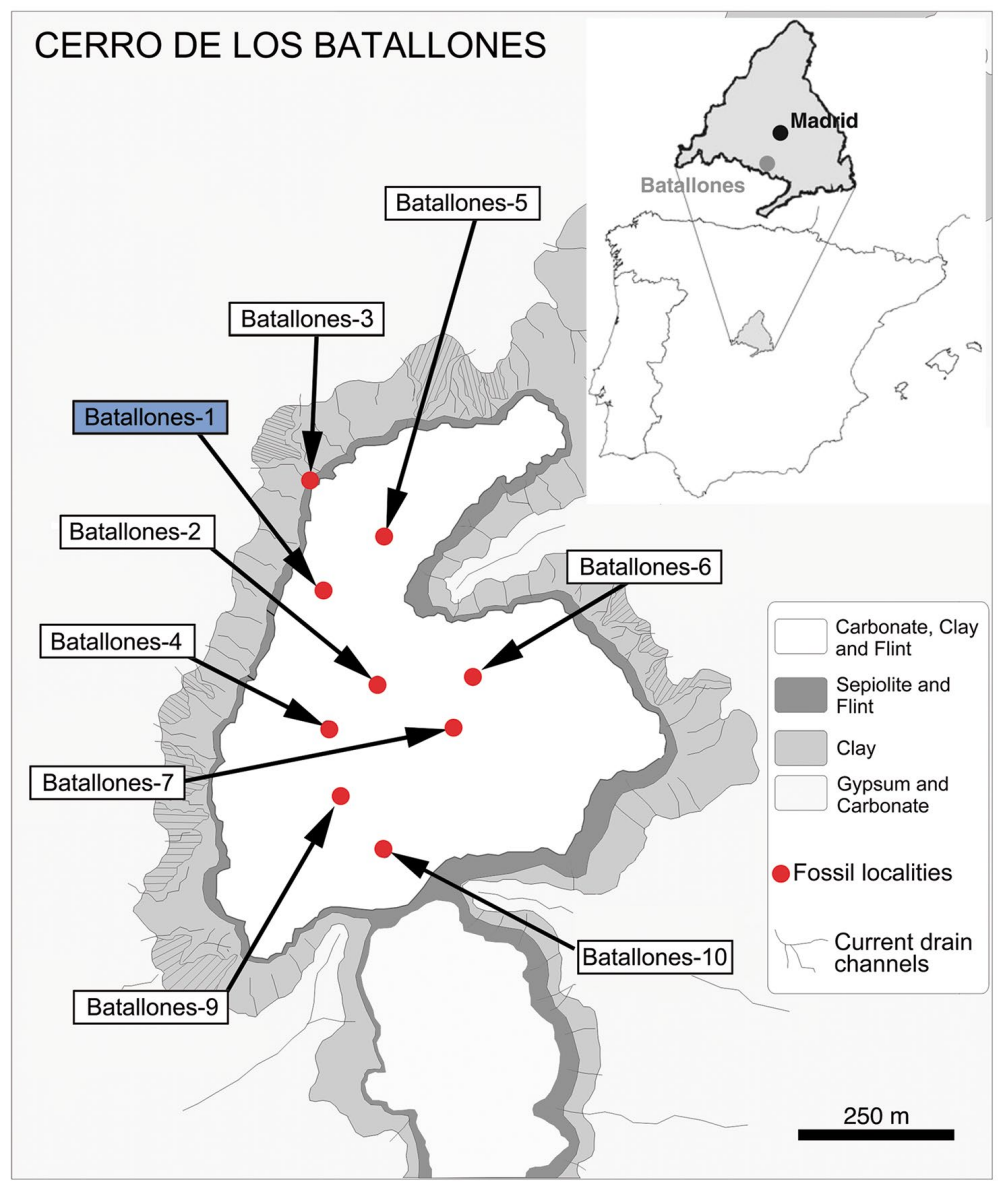
Schematic map of the Cerro de los Batallones paleontological complex showing the location of Batallones-1 fossil site within Spain and the Community of Madrid, and general description of the lithology of the area (taken from [11])

The anatomy of the primitive Smilodontini *Promegantereon ogygia* (Kretzoi, 1938) [1] was virtually unknown until the discovery in 1991 of the Late Miocene (Vallesian, MN 10) fossil locality of Batallones-1 (Madrid, Spain) within a small area that has provided until now nine fossil localities of exceptional interest (Fig. 1). Batallones-1 acted as a natural trap [2] and thus accumulated a huge number of incredibly well-preserved cranial and postcranial fossils of vertebrates, including the most complete sample of *P. ogygia* known to date [3–7]. This site also yielded abundant fossils of another sabre-toothed felid, the much larger, tiger-sized *Machairodus aphanistus* [8] highlighting the relevance of Batallones-1 for the understanding of the evolution of the subfamily Machairodontinae. In fact, these two large felids shared their habitat with two species of small felines, *Pristifelis attica* and *Leptofelis vallesiensis* [9, 10], which makes Batallones-1 one of the Cenozoic fossil sites with the highest diversity of felids.

In previous publications we studied aspects of the functional anatomy of the skull and forelimb of *P. ogygia* [4, 6, 12] describing this machairodontine as an exemple of the mosaic pattern that characterized the evolution of early sabre-toothed felids, as it shows a mixture of derived and primitive features in the mandible, skull and forelimb. For example, the mandible exhibits a large coronoid process, a primitive feature very similar to that of extant pantherines, whereas the mandibular symphysis shows a verticalized rostral part, which insinuates the machairodontine morphology [4]. Concerning the postcranial skeleton, *P. ogygia* had a very robust Mc I (first metacarpal), a large dewclaw, powerful flexor muscles of the elbow, and a scapula built for resisting great biomechanical tensions at the shoulder [6], all derived features typical of the machairodontine killing technique, the so-called canine shear-bite, as initially described in the Pleistocene *Smilodon fatalis* [13]. This hunting method was based on rapidly subduing prey during hunting, in order to apply a shearing bite on the prey’s throat that would cut blood vessels and the trachea, thus producing a rapid kill [14]. This suggested that *P. ogygia* had already developed those anatomical parts closely involved in the deployment of this killing method, whereas other parts remained primitive, subject to less evolutionary selective pressure [4–6]. *Promegantereon ogygia* was part of the first event of diversification of the Machairodontinae, and shared its habitat with the much larger, tiger-sized *Machairodus aphanistus* [8]. Each of them illustrates one of the two main morphotypes developed by this group, Smilodontini and Homotherini, which showed differences in cranial and postcranial anatomy throughout their evolutionary histories since their origins, reflecting their more ambushing or cursorial habits, respectively, although they shared the common killing method, the canine shear-bite [13].

In this work we present the first functional analysis of the hindlimb of *P. ogygia*, which hitherto had remained unknown. Our study shows that, unlike the derived forelimb, the hindlimb was basically primitive, several characters being closer to the early felid *Proailurus lemanensis* than to other more derived sabre-toothed felids. Nevertheless, the pelvis shows a morphology adapted to generate strong propulsive forces during locomotion, as well as to control the lateral movements of the back, which is related to the sabre-toothed hunting method. This study demonstrates mosaic evolution of this early form of Late Miocene machairodontine, and emphasizes interest in studying more primitive forms, such as the Middle Miocene genus *Pseudaelurus*, something that will be the focus of our future research.

## Materials and methods

The fossils of *Promegantereon ogygia* studied here belong to the sample from the Late Miocene (Vallesian, MN 10) palaeontological site of Batallones-1 (Madrid, Spain), temporarily housed at the collections of the Museo Nacional de Ciencias Naturales-CSIC (Madrid, Spain). These fossils were included in the PhD of Salesa [15], but here they are described and discussed in depth for the first time. Comparisons with extant carnivorans were made using specimens from the collections of the Museo Anatómico de la Universidad de Valladolid (Valladolid, Spain), and Museo Nacional de Ciencias Naturales-CSIC (Madrid, Spain), which provided complete skeletons of the felines *Leopardus wiedii*, *Acinonyx jubatus* and *Puma concolor*, the pantherines *Panthera leo*,* Panthera pardus*, *Panthera onca*, and *Panthera uncia*, and the viverrid *Genetta genetta*. Comparisons with other extinct members of Felidae, such as *Panthera atrox*, *Smilodon fatalis*, *Smilodon gracilis*, *Smilodon populator*, *Pseudaelurus quadridentatus*, *Megantereon cultridens*, *Proailurus lemanensis*, and *Homotherium latidens* were made using unpublished images stored by the authors, as well as published data (see citations in the corresponding sections).

**Fig. 2:**
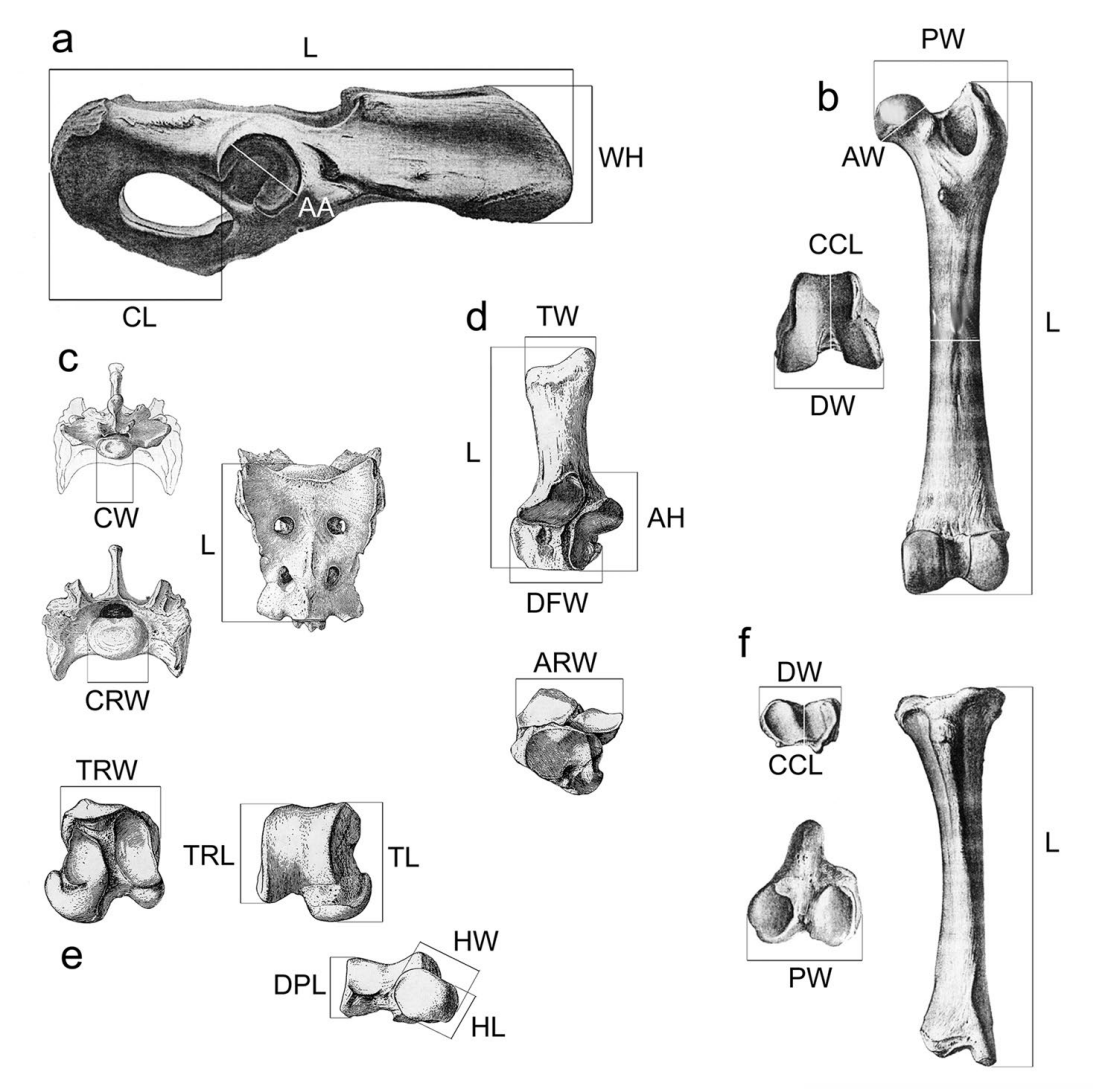
Measurements for each studied postcranial element; **a**, coxae; **b**, femur; **c**, sacrum; **d**, calcaneus; **e**, talus; **f**, tibia. (AA: acetabular length; AH: articular proximodistal height; ARW: articular width; AW: articular width; CCL: craniocaudal length; CL: caudal length; COW: cochlear width; CW: caudal width; CRW: cranial width; DFW: distal facet width; DPL: dorsoplantar length; DW: distal width; HL: head length; HW: head width; L: proximodistal length; TL: total length; TRL: trochlear length; TRW: trochlear width; TW: tubercle width; PW: proximal width; WH: iliac wings height)

The anatomical descriptions follow the terminology used by Barone [16], Evans [17], and ICVGAN [18]. The measurements were taken with a digital calliper and are shown in Fig. 2; Tables 1, 2, 3, 4, 5 and 6.

## Anatomical descriptions

### Coxae

**Fig. 3:**
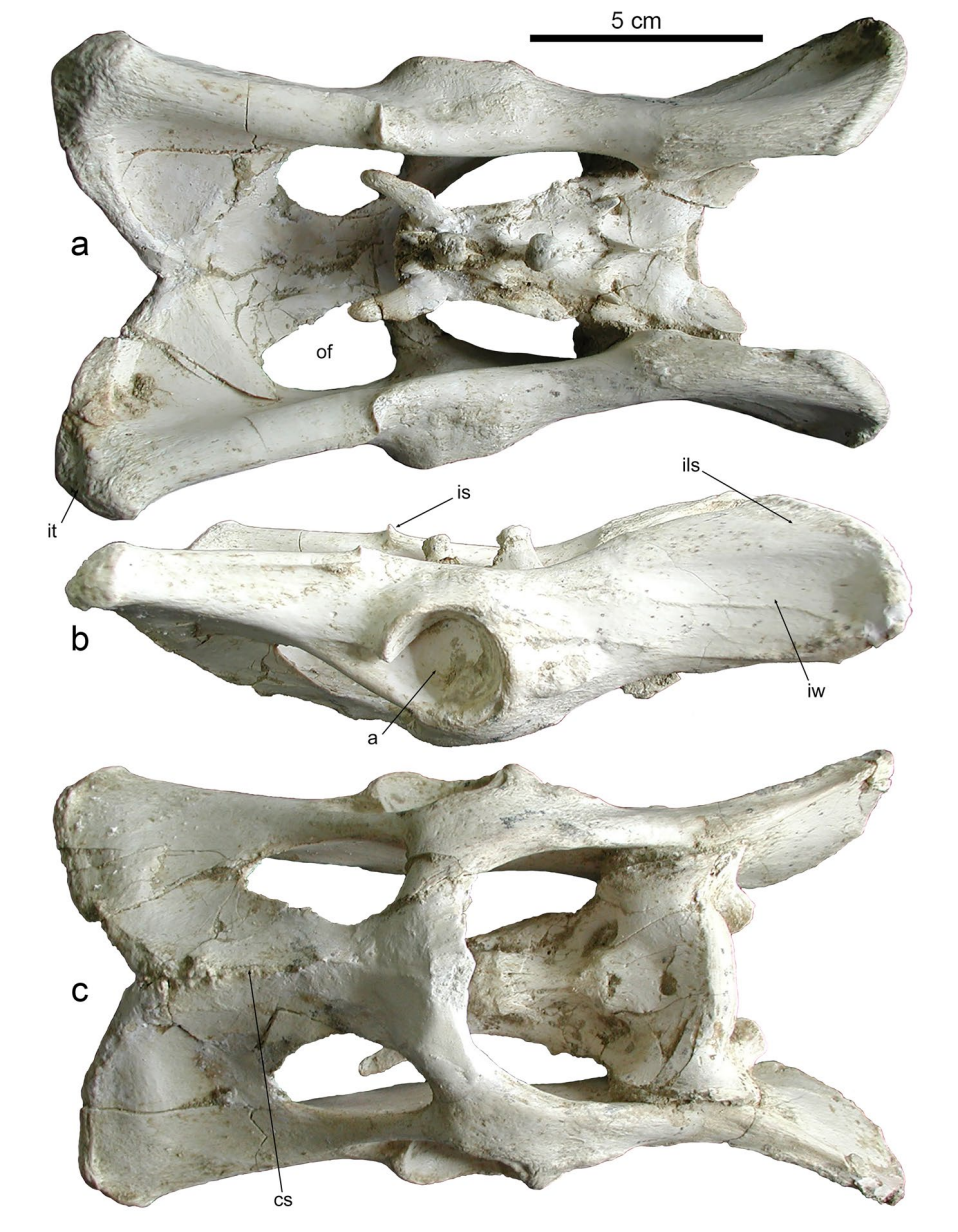
B-4326, pelvis and sacrum of *Promegantereon ogygia*; **a**, dorsal view; **b**, right lateral view; **c**, ventral view. (a: acetabulum; cs: crista symphysialis; ils: iliac spine; is: ischiatic spine; it: ischiatic tuberosity; iw: ilium wings; of: obturator foramen)

The size and overall morphology of the coxae of *Promegantereon ogygia* are similar to those of similarly-sized felines and pantherines, although a detailed observation reveals a number of differences with functional implications. In dorsal view (Fig. 3a), the dorsal border of the coxae of *P. ogygia* is thick, smooth, and quite straight, with its cranial third slightly curved in lateral direction. *Panthera leo*, *Pa*. *atrox*, *Pa. pardus*,* Pa. uncia* and *Pu*. *concolor* show a very straight dorsal border, with the plane of the ilium wing almost parallel to the sagittal plane, whereas in *P. ogygia*, *H. latidens* and *S. fatalis* this plane is clearly dorsolaterally inclined, with the ventral border of the wing being laterally projected. Interestingly, *Pa. onca*, the most robust of the pantherines, exhibits a similar curvature as these latter sabre-toothed species. A marked ischiatic spine is located at the level of the caudal border of the acetabulum in *P. ogygia*, separating the attachment areas for the mm. gemelli (caudal to the spine) and m. gluteus profundus (cranial to the spine). The ischiatic spine is more cranially located in *Pa. leo*, *Pa. atrox*, *Pa. onca*, *Pa. pardus*,* Pa. uncia* and *Pu*. *concolor*, which produces a relatively longer attachment area for the mm. gemelli. The caudal vertex exhibits a rough ischiatic tuberosity for the attachment of the m. gluteus superficialis, m. biceps femoris, m. semimembranosus, and m. semitendinosus, as well as for the attachment of the lig. sacrotuberale.

In lateral view, the wings of the ilium of *P. ogygia* show a subrectangular shape (Fig. 3b), with their cranial border displaying a gentle dorsoventral inclination. On the middle part of the wings, there is a marked depression for the attachment of the m. gluteus accessorius, whereas the dorsal border is rough and thickened for the attachment of the m. gluteus medius. Along the ventral border of the ilium, there is an attachment surface for the m. iliacus, which is smooth but with slightly ridged margins. Cranial to the acetabulum, on the lateral face, there are a couple of tuberosities separated by a rough depression; one tuberosity is located dorsally to the other, and is the attachment surface of the m. articularis coxae, whereas the second bulge, ventral to the former and much larger, provides an attachment area for the m. rectus femoris. The ischium is elongated with a rough ischiatic tuberosity and an elliptic obturator foramen. When comparing this morphology to that of felines and pantherines, the ilium of *P. ogygia* is craniocaudally relatively shorter than that of *Pa. leo*, *Pa. atrox*, *Pa. pardus*, *Pa. onca*, *Pa. uncia* and *Pu. concolor*, with a less marked ilium neck, whereas the ischium is as long as those of these mentioned taxa; besides, the dorsal margin of the ilium of *P. ogygia* does not show the dorsocaudal iliac spine observed in these later taxa, and also in *H. latidens*; interestingly, in *S. fatalis* this spine is much more marked than in any other compared felid, protruding dorsally as a rough tuberosity. In these latter sabre-toothed felids, the ilium is relatively shorter than those of similarly-sized felines and pantherines, as well as the ischium.

In medial view, the coxae of *P. ogygia* shows a wide, round and irregular surface for articulation to the sacrum. Cranial to this surface there is a rough area for the attachment of the m. erector spinae. On the medial surface of the ischium, surrounding the obturator foramen, there is a smooth surface for the attachment of the m. obturatorius internus. There are no notable differences among the compared species.

In ventral view (Fig. 3c), the symphysis pelvis of *P. ogygia* shows a marked and rough crista symphysialis, part of the attachment area of the m. gracilis. The obturator foramina are elliptical, with their major radii almost craniocaudally oriented; ventrocaudally to each obturator foramen, the attachment surfaces for the m. obturatorius externus and m. gracilis show a smooth, poorly marked surface. On the ventral border of the ischiatic tuberosity there is a rough surface for the attachment of m. quadratus femoris. There are no relevant differences in this view among the compared species.

**Table 1:** Measurements in mm of the coxae of *Pr. ogygia* from Batallones-1

Measurement	*n*	Average	SD	Maximum	Minimum
L	10	177.43	9.21	188.35	163.95
AA	18	23.51	1.59	26.38	20.60
CL	16	64.75	3.22	69.93	58.69
WH	14	37.85	4.59	47.11	31.42

### Sacrum and caudal vertebrae

**Fig. 4:**
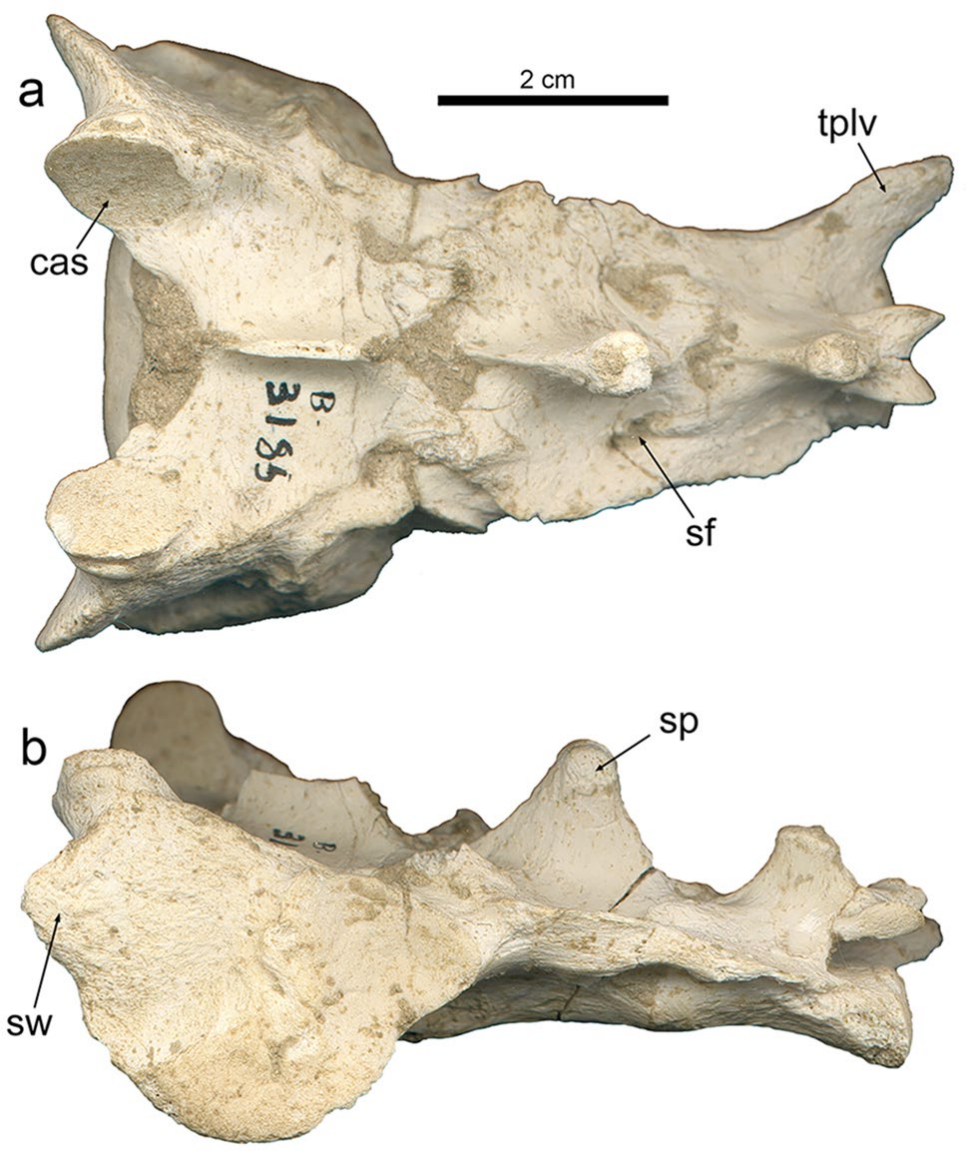
B-3185, sacrum of *Promegantereon ogygia*; **a**, dorsal view; **b**, left lateral view. (cas: cranial articular surface; sf: sacral foramen; sp; spinous process; sw: sacral wing; tplv: transverse process of the last sacral vertebrae)

The overall shape of the sacrum of *P. ogygia* is slightly different from that of the extant compared large felines and pantherines. The former has a craniocaudally longer sacrum, with a laterally narrower caudal end (Figs. 3a and 4), more similar to that of lynxes than to that of pantherines. This means that the first caudal vertebrae of *P. ogygia* are also narrower than those of the compared felines and pantherines, and thus the tail was shorter, similar to those of more derived sabre-toothed felids such as *H. latidens*,* Me. culdridens* and *S. fatalis*, but also resembling those of extant felines such as *C. caracal*,* L. serval* and *Lynx* spp. Interestingly, the sacra of both *S. fatalis* and *Me. cultridens* are not triangular [19, 20], unlike that of *P. ogygia*, but almost rectangular, similar to those of pantherines, due to the relatively wide caudal end in comparison to the Batallones-1 sabre-toothed felid. In dorsal view, the sacrum of *P. ogygia* (Fig. 4a) shows round articular cranial processes, cranially projected sacrum wings, two sacral foramina on each side, and a thin and laterocaudally projected transverse process of the last sacral vertebra. The extant compared felines and pantherines, *Pa. pardus*, *Pa. onca*, *Pa. uncia* and *Pu. concolor* show no significant differences in this view. In *S. fatalis* the transverse processes of the last sacral vertebra are clearly shorter and less projected. In lateral view (Fig. 4b), the spinous processes of *P. ogygia* are subtriangular, and decrease in height craniocaudally; the one of the first sacral vertebra is broken in all the specimens, whereas that of the second is triangular and ends in a rough round bulge; finally, the spinous process of the third sacral vertebra is subtriangular, with a rough, round and dorsally flattened bulge. The compared felines and pantherines show this same pattern, although the spinous processes have a less massive tip, with *Pu. concolor* showing relatively higher processes. In *S. fatalis* the spinous processes have a massive and rough tip, very similar to those of *P. ogygia*.

The caudal vertebrae of *P. ogygia* show interesting differences with those of large felines and pantherines. The first four caudal vertebrae of *P. ogygia* have strong cranial and caudal articular processes, and a very reduced spinous process, limited to a small dorsal thickening. The transverse processes are thin, triangular, and caudally oriented; their cranial border is quite straight and lacks any notch. This morphology is very similar to that of the first four caudal vertebrae of *S. fatalis*, *H. latidens*, and *Ly. pardinus*. On the contrary, in *A. jubatus*, *P. leo*, *P. atrox*, *P. onca*, *P. pardus*, and *Pu. concolor*, the transverse processes have a clear notch on their cranial border near their junction with the vertebral body; unlike in *P. ogygia*, these processes are not triangular but rather sub-rectangular. The following caudal vertebrae of *P. ogygia* show a very simple morphology, being reduced to a cylindrical and elongated vertebral body with strongly reduced cranial and caudal articular processes.

**Table 2:** Measurements in mm of the sacrum of *Pr. ogygia* from Batallones-1

Measurement	*n*	Average	SD	Maximum	Minimum
CRW	9	27.56	1.93	30.74	24.28
CW	6	13.30	0.49	13.80	12.61
L	8	68.48	3.68	74.03	62.02

### Femur

**Fig. 5:**
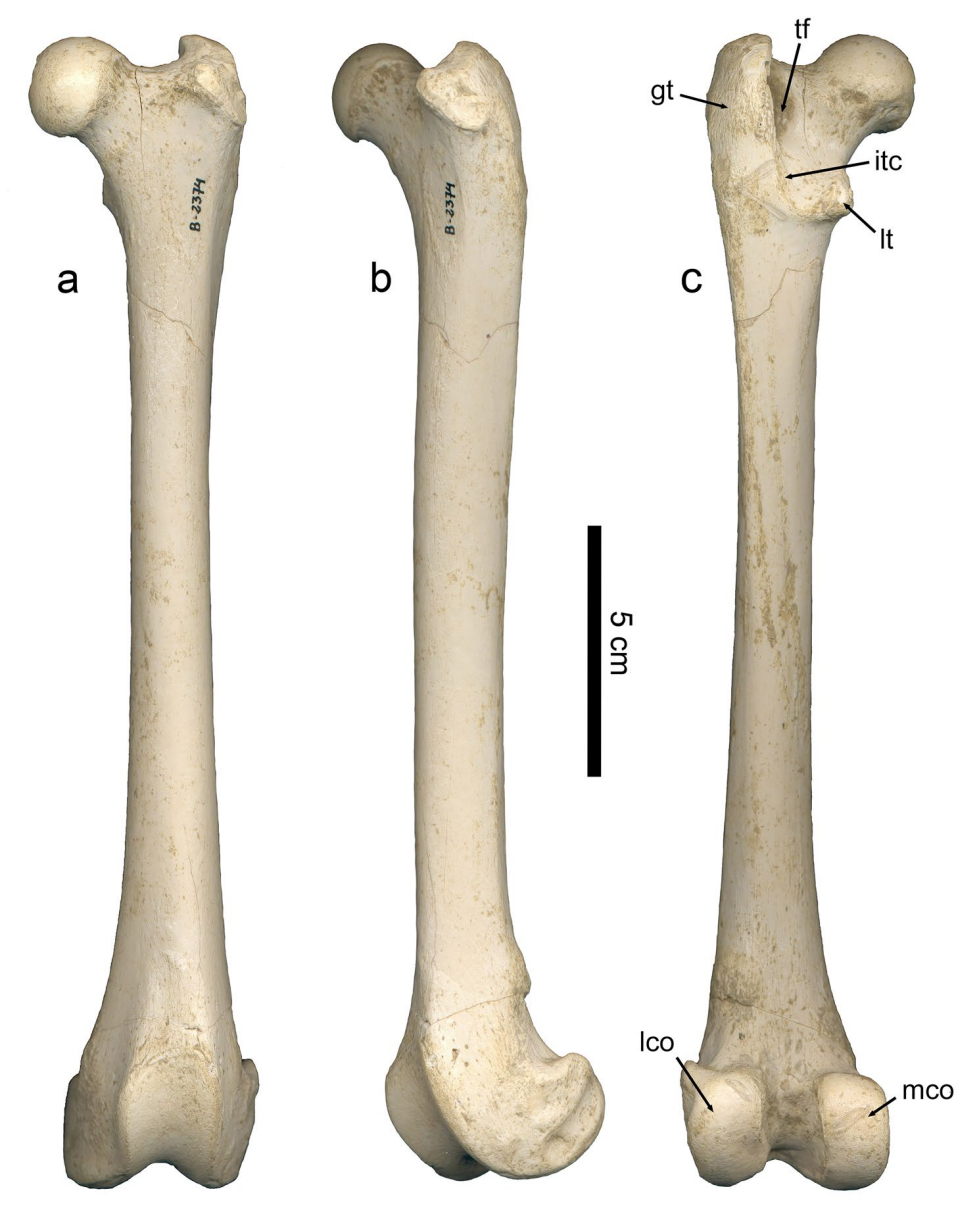
B-2374, left femur of *Promegantereon ogygia*; **a**, cranial view; **b**, lateral view; **c**, caudal view. (gt: greater trochanter; itc: intertrochanteric crest; lco: lateral condyle; mco: medial condyle; lt; lesser trochanter; tf: trochanteric fossa)

The Batallones-1 sample includes nine right and 12 left femora of *P. ogygia*, some of them fairly complete. In caudal view, the femoral head is projected proximomedially and shows a short but well-developed neck; the proximal border of the head is located at the same level as the proximal border of the greater trochanter (Fig. 5a); this morphology is very similar to that of *H. latidens*, *Pa. pardus*, *Pa. onca*, *Pa. uncia* and *Pu. concolor*, whereas in *Me. cultridens* and *S. fatalis*, the proximal border of the greater trochanter slightly surpasses the level of the femoral head. In lateral view, the femur of *P. ogygia* has a very smooth gluteal tuberosity contacting proximally the greater trochanter, with a soft ridge that can be absent in some individuals (Fig. 5b); in the rest of the compared large felines and pantherines, as well as in the machairodontines *H. latidens*, *Me. cultridens* and *S. fatalis*, this tuberosity shows a rougher surface, with a more marked cranial ridge. Distal to the gluteal tuberosity, on the lateral margin, there is a smooth ridge, similarly developed in *P. ogygia* and the compared species, which continues distally to the middle of the diaphysis; it serves as an attachment surface for the m. adductor magnus. In caudal view (Fig. 5c), the greater trochanter is mediolaterally narrower in *P. ogygia* when compared to those of *H. latidens*, *Me. cultridens*,* S. fatalis*, and the extant large felines and pantherines (especially those of *Pu. puma* and *Pa. uncia*), whereas in lateral view, only the greater trochanter of *Pa. onca*, *H. latidens* and *S. fatalis* are craniocaudally longer. On the proximal tip of the greater trochanter, the attachment areas for the m. gluteus profundus, m. gluteus accessorius and m. gluteus medius show a similar pattern in all the compared taxa, with the attachment areas of the two latter being located proximal to each other, whereas that of m. gluteus profundus being developed just distal to the two others. In caudal view (Fig. 5c), the proximal epiphysis of *P. ogygia* shows a moderately excavated trochanteric fossa, a ridged intertrochanteric crest, and a caudomedially projected lesser trochanter; in the compared felines and pantherines, the lesser trochanter is similarly developed and located (there are just subtle differences in the orientation), but the trochanteric fossa is markedly deeper in all the taxa; in *S. fatalis*, the lesser trochanter is relatively smaller than those of the rest of the taxa, and more centrally located; besides, the trochanteric fossa is deeper and lateromedially wider in *S. fatalis* than in the other compared felids, and the lateral border of the greater trochanter is more inflated and laterally expanded.

**Table 3:** Measurements in mm of the femora of *Pr. ogygia* from Batallones-1

Measurement	*n*	Average	SD	Maximum	Minimum
L	13	232.35	4.35	239.40	225.55
PW	21	46.68	2.27	51.42	41.95
DW	11	41.15	1.37	42.66	37.36
CCL	9	27.70	1.55	29.72	25.41
AW	20	22.55	0.98	24.85	21.13

In the distal epiphysis of *P. ogygia* and the compared species, both the lateral and medial condyles show a similar mediolateral width; in caudal view (Fig. 5c), the condyles show a similar craniocaudal development, they being separated by a wide intercondyloid fossa. The medial condyle shows a slight medial expansion in *P. ogygia*, which is more evident in the compared extant felids, especially in *Pa. uncia* and *Pa. onca*, but also in the machairodontines *H. latidens* and *S. fatalis*. In caudal view, the medial condyle is quite parallel to the proximodistal axis of the femur, whereas the lateral one is slightly laterally inclined, with its proximolateral vertex showing a proximolateral expansion. In this caudal view, it is evident that both the medial condyle and the intercondyloid fossa of *P. ogygia* and *S. fatalis* are mediolaterally narrower than those of *Pa. pardus*, *Pa. onca*, *Pa. uncia* and *Pu. concolor*. Also, in caudal view, in all the compared species, including *P. ogygia*, both condyles reach the same level distally. In cranial view, the trochlea of *P. ogygia* is relatively lateromedially narrower than those of *Me. cultridens*,* Pa. pardus*, *Pa. onca*, *Pa. uncia* and *Pu. concolor*, whereas those of *S. fatalis* and *H. latidens* are even wider than those of the former. Finally, in lateral and medial views, both the craniocaudal length of the distal epiphysis, and the curvature of the femoral trochlea of *P. ogygia* are very similar to those of the other compared species. 

### Tibia

**Fig. 6:**
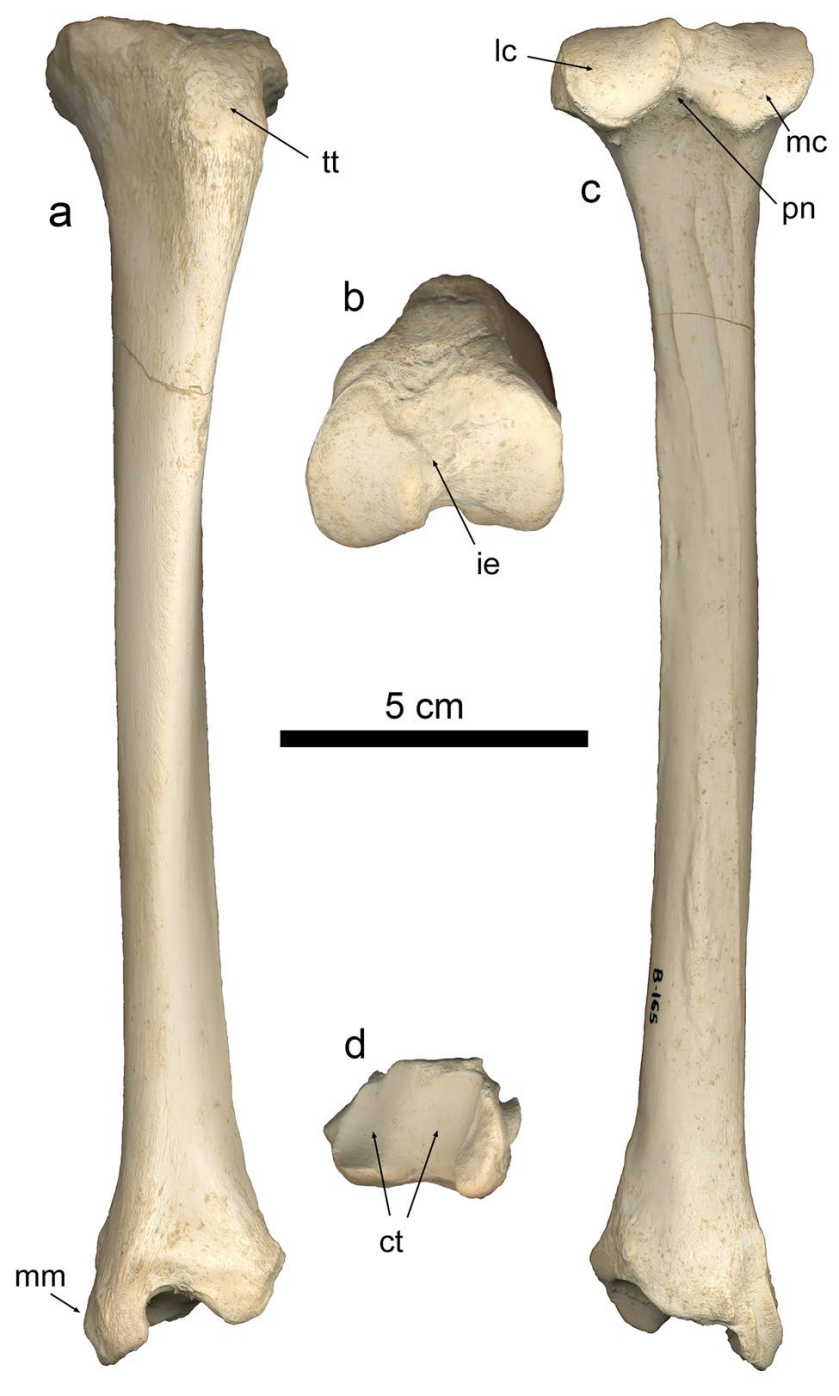
B-165, left tibia of *Promegantereon ogygia*; **a**, cranial view; **b**, proximal view; **c**, distal view; **d**, caudal view. (ct: cochlea tibiae; lc: lateral condyle; mc: medial condyle; mm: medial maleolus; tt, tibial tuberosity; pn, popliteal notch)

The Batallones-1 sample includes 40 tibiae of *P. ogygia*, 23 right and 17 left. The tibia is slender in overall view, with a more or less straight diaphysis and a caudally curved proximal epiphysis (Fig. 6a). The proximal epiphysis of *P. ogygia* shows a triangular-shaped proximal surface, with the two articular condyles (medial and lateral) separated by a rough and lateromedially wide cranial intercondyloid area and a much narrower caudal intercondyloid area (Fig. 6b); these condyles are irregularly round, the lateral one being slightly more projected caudally than the medial one. In *Pa. pardus*, *Pa. onca*, *Pa. uncia* and *Pu. concolor* the articular surface of the medial condyle shows an elliptical lateromedially oriented expansion, which seems to expand the attachment facet of the medial meniscus and its corresponding transverse ligament. Interestingly, this facet is completely absent in *P. ogygia*, *Me. cultridens*, *H. latidens* and *S. fatalis*, whereas in the primitive felid Proailurus lemanensis, the facet is present although it is less laterally extended than in the compared extant felids. In both the cranial and caudal views, the lateral condyle in *P. ogygia* is slightly more proximally projected than the medial one, and the medial condyle shows a concave proximal border, which also contributes to its lesser proximal expansion (Fig. 6a, c). In *Pa. pardus*, *Pa. onca*, *Pa. uncia* and *Pu. concolor* the medial condyle shows an almost flat proximal surface, which is very evident from the caudal view. In proximal view, the cranial border develops a strong and laterally projected tibial tuberosity, with a marked notch on its lateral margin for the passage of the tendon of the m. extensor digitorum longus, and a rough lateral small facet for the attachment of the m. biceps femoris. The tibial tuberosity in *P. ogygia* and the compared pantherines and felines has a smooth proximal surface separated by a ridge from the cranial border, which runs distally until the middle of the cranial margin of the diaphysis. In lateral view, the epiphysis of *P. ogygia* hows a relatively larger articular facet for the fibula compared with those of the extant felids; also, in this view, the caudal border is less caudally projected in the felid from Batallones-1 than in the compared felines and pantherines. In medial view, the round facet for the attachment of the m. semimembranosus located at the proximocaudal border shows no differences among all the studied felids.

**Table 4:** Measurements in mm of the tibia of *Pr. ogygia* from Batallones-1

Measurement	*n*	Average	SD	Maximum	Minimum
L	21	215.51	5.64	223.85	205.45
DW	30	31.88	1.81	34.50	28.64
CCL	32	18.40	1.04	20.59	16.00
PW	31	43.45	2.47	48.31	38.69
LT	27	27.63	2.08	30.60	20.28

The diaphyses of *P. ogygia* and the compared felines and pantherines are slightly mediolaterally flattened on its proximal half, whereas the distal half has an almost round section. Also, the proximal half is slightly laterally inclined in cranial view, and caudally in both lateral and medial views; the distal half is quite straight. In caudal view, the proximal half of the diaphysis of *P. ogygia* shows a couple of parallel crests that run distally from the middle surface of the epiphysis to the medial margin of the diaphysis (Fig. 6d), where the most laterally located extends distally by means of a soft ridge. In extant large felines and pantherines, this lateral crest separates the attachment area of the m. flexor digitorum lateralis (= m. flexor hallucis longus) from the one of the* m. tibialis caudalis*, whereas the medial crest separates this latter from the m. flexor digitorum medialis and m. popliteus [16, 21]. In the compared felines and pantherines, as well as in *S. fatalis* and *H. latidens*, the area for the *m. tibialis caudalis* is relatively wider and more excavated than those of *P. ogygia* and *M. cultridens*, especially in the case of *Pa. pardus* and *Pa. onca*, probably due to the presence of a larger muscle.

The distal epiphysis of *P. ogygia* strongly resembles that of the compared felines and pantherines, although there are some differences. In cranial view, the transition from the diaphysis to the distal epiphysis in *P. ogygia* is smooth, without the obvious constriction observed in the compared felines and pantherines. On the lateral margin, there is a triangular facet for the fibula, with a similar position in all the compared species. On the medial face of the distal epiphysis of *P. ogygia* there are three crests, which are located on the caudolateral border and delimitate two proximodistally elongated grooves. The central crest is less developed and almost absent in some of the specimens (for example, BAT-1’05 F6-42), but is well developed in others (BAT-1’24 92), showing that this character is subject to great variability. No significant differences are observed when this area is compared in the studied felines, pantherines and machairodontines. In distal view, the cochlea tibiae shows two parallel, oblique grooves for articulating the trochlea of the talus. There are no remarkable differences between *P. ogygia* and the compared species.

### Calcaneus

**Fig. 7:**
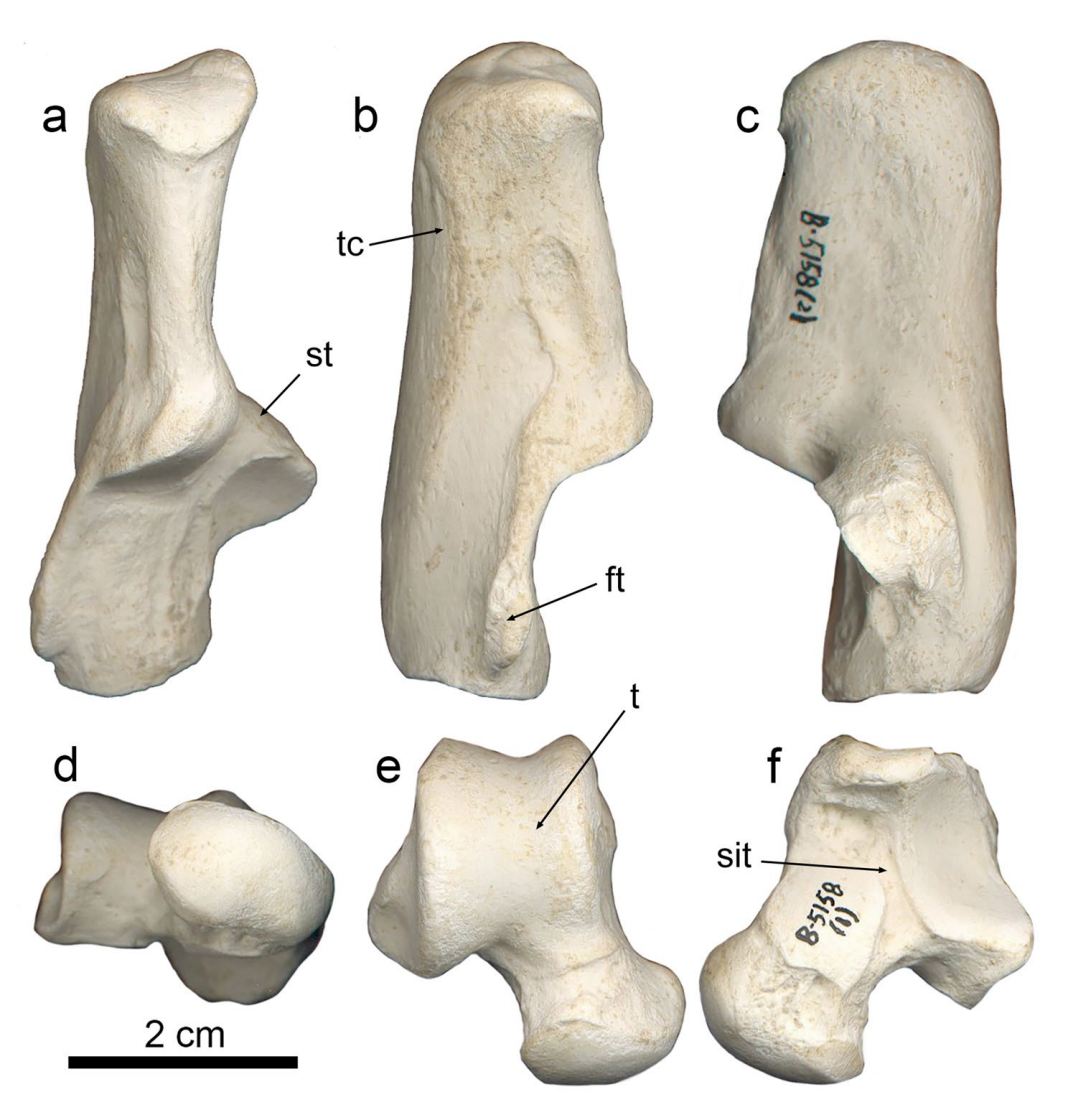
B-5158 (2), right calcaneum, and B-5158 (1), right talus of *Promegantereon ogygia*; **a**, dorsal view; **b**, lateral view; **c**, medial view; **d**, distal view; **e**, dorsal view; **f**, plantar view. (ft: fibular tubercle; sit: sinus tarsi; st: sustentaculum tali; t: trochlea; tc: tuber calcanei)

The calcanei of *P. ogygia* and the compared felines and pantherines show similar overall morphology and proportions. In lateral view (Fig. 7b), the fibular tubercle is proximodistally elongated, with a marked central groove and ridged dorsal and plantar borders; it is located very close to the distal border of the bone, and it is strongly laterally projected; some specimens (e.g., B-4818c) show a reduced fibular tubercle, but this structure generally shows a larger size among the sample from Batallones-1 than in the latter specimen. Among the compared extant felines and pantherines, the fibular tubercle shows a similar morphology to that of *P. ogygia*, although it is less laterally projected, and the central groove is shallower; this latter feature is more evident in *Pa. onca*, which has a round fibular tubercle that lacks the central groove. In *S. fatalis* the fibular tubercle is slightly less laterally projected than in *P. ogygia*, but it is thicker and with a shallower central groove, whereas in the calcaneus of *Me. cultridens*, the fibular tubercle is even more laterally projected than in *P. ogygia*, and has a marked central groove. In contrast, in *H. latidens*, the fibular tubercle is reduced to a small bulge. Finally, in *Pr. lemanensis*, the fibular tubercle is round, with a marked central groove, and it is proportionally larger than those of the compared sample. In *P. ogygia* the dorsal border of the fibular tubercle develops a proximally elongated ridge until the middle of the tuber calcanei, due to the existence of an excavated area for the attachment of the m. quadratus plantae [21]; this attachment area is much less excavated in *Pa. pardus*, *Pa. onca*, *Pa. uncia* and *Pu. concolor*, but its proximodistal development is reduced in *Pa. pardus*, *Pu. concolor* and *Pa. onca*, especially in this latter, whereas in *Pa. uncia* the attachment area has almost the same proximodistal length as in *P. ogygia*. In *Me. cultridens*, the attachment area for the m. quadratus plantae shows a similar extension and depth to those of *Pa. pardus* and *Pa. onca*, whereas in *S. fatalis* and *H. latidens* there is no clear attachment area, probably due to the extreme reduction of this muscle. In *Pr. lemanensis*, the attachment area for the m. quadratus plantae is even more elongated than that of *P. ogygia*. In the proximodorsal margin of the attachment area for the m. quadratus plantae there is an elliptic rough area for the attachment of the lig. calcaneofibularis, which plays an important role in the stabilization of the ankle joint, generating a tension force in the tendons of the m. fibularis longus and m. fibularis brevis during the inversion and dorsiflexion of the foot [22–24]; this scar is more distally extended in *Pa. pardus*, *Pa. uncia*, *Pu. concolor* and *Pa. onca*, but also in the sabre-toothed felids *S. fatalis*, *Me. cultridens* and *H. latidens*. In contrast, in *Pr. lemanensis*, this facet is located in a very similar position to that of *P. ogygia*.

**Table 5:** Measurements in mm of the calcaneus of *Pr. ogygia* from Batallones-1

Measurement	*n*	Average	SD	Maximum	Minimum
L	35	63.14	2.50	67.42	58.03
ARW	24	26.26	1.79	30.12	23.75
DFW	35	17.26	1.15	15.18	19.77
AH	32	25.25	1.67	30.11	22.31
TW	33	17.00	1.09	19.22	14.98

In its medial face (Fig. 7c), the sustentaculum tali is strongly medially projected in *P. ogygia* and the compared sample. This structure has a rounded medial talar facet that continues distally by a narrow and long facet until the distomedial border of the talar surface, where it widens in a distal round small facet; this facet is dorsomedially oriented in *P. ogygia*, *Pa. pardus*, *Pa. onca* and *Pa. uncia*, whereas in *Pu. concolor* it is medially oriented. The medial talar facet is round in *S. fatalis* and *H. latidens*, but as the calcaneus is relatively proximodistally shorter in these two genera, the distal expansion is short and wide, and the distal facet is just a continuation of this expansion.

The tuber calcanei is relatively slender and mediolaterally flattened in *P. ogygia*, *Me. cultridens* and the compared felines and pantherines. In *S. fatalis*, although the calcaneus is relatively proximodistally shorter, the tuber keeps the same proportions as in *P. ogygia*, and is the talar area which is clearly shortened. In *H. latidens*, the tuber calcanei is relatively shorter and wider than those of the rest of the compared sample. In dorsal and plantar views, the medial tubercle of the tuber is more proximally projected than the lateral one in *P. ogygia* and the rest of the compared sample, including the machairodontines, although the difference is even more evident in *Pu. concolor*, *Me. cultridens*, and *S. fatalis*, as the medial tubercle is markedly projected in proximal direction.

In plantar view, the groove for the tendon of the m. flexor digitorum lateralis, located on the plantar surface of the sustentaculum tali, is similarly marked in all the species. On the distal end of this plantar face, a rough scar for the lig. plantare longum, very similar in all the species, is observed.

In proximal view, the tuber calcanei shows an elliptical shape in all the species, slightly dorsoplantarly elongated, and with the medial tubercle being larger than the lateral one. In distal view, the articular facet for the cuboid is D-shaped in all the compared species.

### Talus

The overall morphology and proportions of the talus of *P. ogygia* are quite similar to those of *Pa. onca*, *Pa. pardus*, *Pa. uncia* and *Pu. concolor*. The trochlea is square in dorsal view, with a shallow central groove that divides it into two asymmetrical lips (Fig. 7e), as the medial one is slightly narrower than the lateral one. Also, the lateral lip is slightly more projected distally, even though the distal border of the medial lip expands distally onto the neck; this is observed in the tali of the compared felines and pantherines, as well as in those of *H. latidens*, *Me. cultridens* and *S. fatalis*. In proximal view, the lateral and medial lips are similarly projected dorsally. The proximal surface of the trochlea of *P. ogygia* shows a shallow groove delimitating the lateral margin of the medial lip, and a small talar foramen. The presence of this foramen is typical of felids [25, 26], and thus it is present in all the compared species; it is in fact remarkable in *S. fatalis*, in which this foramen can be much wider than in other felids. Nevertheless, in the proximal view of the trochlea, it is noteworthy that this foramen is located more dorsally in *P. ogygia* than in the extant compared felines and pantherines. In plantar view (Fig. 7f), the talus of *P. ogygia* shows a relatively narrow sulcus tali that separates two large articular facets for the calcaneus: (i) the ectal face, laterally located, rectangular and strongly concave, and (ii) the sustentacular facet, medial to the former, which is round, convex, and occupies most of the plantar surface of the neck; in some specimens (e.g., B-3881) the sustentacular facet reaches the distal articular surface of the talus head (navicular facet) through a narrow projection, whereas in other specimens the expansion is short and does not connect with the head facet (e.g., BAT-1’02 F4-53), or is completely absent in others (e.g., B-4818d). In the compared extant felines and pantherines, only in *Pa. pardus* is the sustentacular facet not connected to the navicular facet. Nevertheless, as suggested by the studied sample of *P. ogygia*, this condition seems highly variable, and has been described in many other species of extant felines and pantherines [10]. The talus neck of *P. ogygia* is as long as those of *Pa. pardus*, *Pa. onca*, *Pa. uncia* and *Pu. concolor*, as well as that of *Me. cultridens*. Merriam and Stock, [20] described a great variability in the length of the talus neck of *S. fatalis* from Rancho La Brea, with some specimens having necks of a similar length to those of *Pa. onca*, whereas others exhibit markedly shorter necks.

**Table 6:** Measurements in mm of the talus of *Pr. ogygia* from Batallones-1

Measurement	*n*	Average	SD	Maximum	Minimum
HL	39	12.80	0.80	14.65	11.16
HW	39	19.27	0.97	21.31	16.94
DPL	31	16.46	1.09	18.14	14.05
TRL	29	24.78	1.32	27.17	21.69
TRW	29	26.22	1.33	29.31	23.42
TL	29	35.38	1.77	37.76	31.18

Finally, in dorsal view, the head of the talus of *P. ogygia* has a round distal margin, with a marked to very soft (depending on the specimens) ridge separating it from the neck; among the compared sample of felines and pantherines, as well as in *S. fatalis*, *Me. cultridens* and *H. latidens*, this ridge shows the same variation observed in *P. ogygia*, even between both sides of the same individual. In distal view, the head of the talus of *P. ogygia* is elliptical (Fig. 7d), with its major axis forming an angle of around 30º with the mediolateral axis of the bone. This orientation is very similar in the compared felines, pantherines and machairodontines, although the head is round rather than elliptical in the former and *S. fatalis*.

## Functional inferences of the hind limb anatomy of *Promegantereon ogygia*

### Coxae

**Fig. 8:**
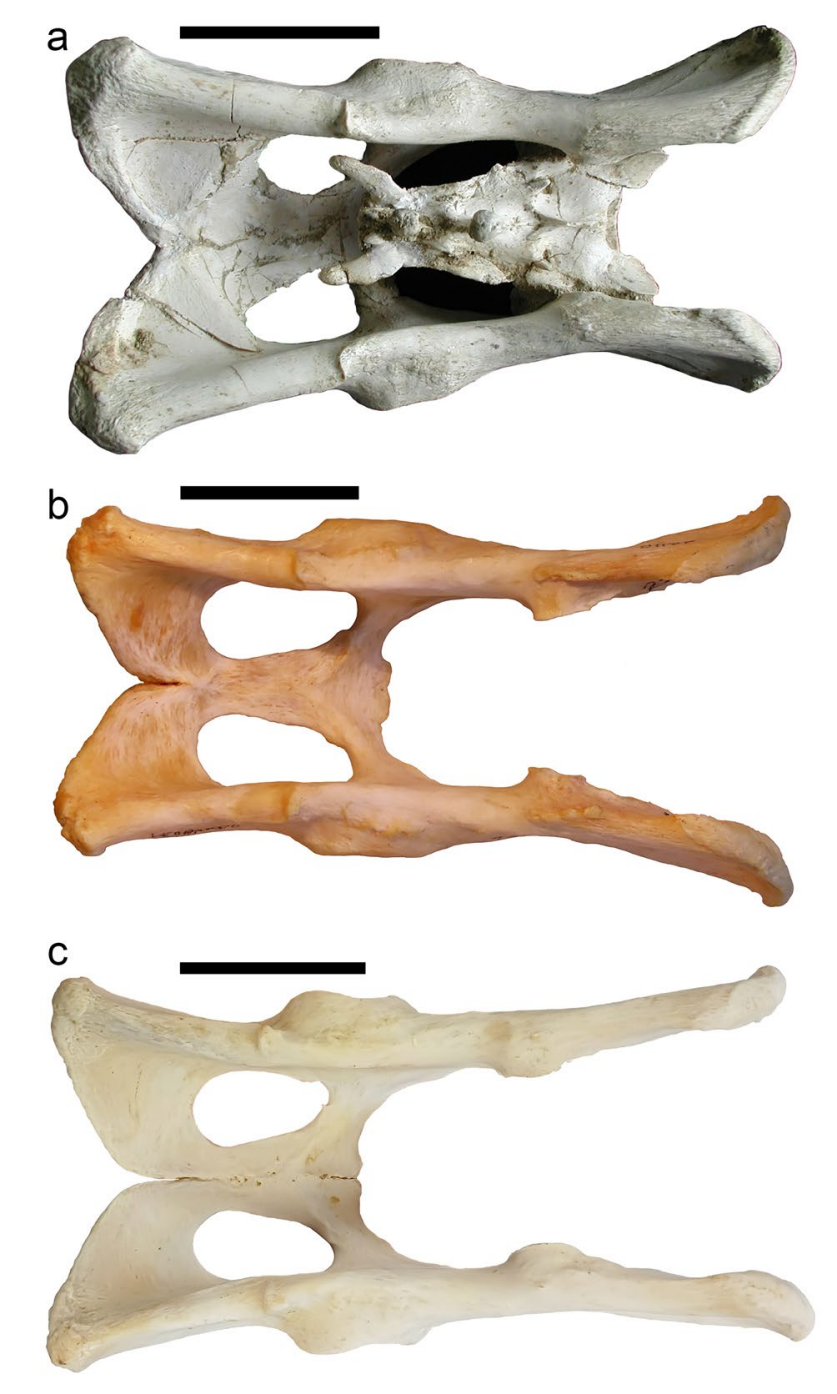
Dorsal view of the pelvis of the compared species of Felidae: **a**, B-2561, *Promegantereon ogygia* (with the articulated sacrum); **b**, MNCN-21,566, *Panthera uncia*; **c**, MAV-2240, *Panthera pardus*. Each bar represents 5 cm

Both the pelvis and lumbar vertebral series of the most derived sabre-toothed felids are relatively craniocaudally shorter than those of the extant large felines and pantherines [27–29]. Nevertheless, the development of this character in the early machairodontines was unknown, due to the absence of fossils of these forms. The plane of the iliac wings of *P. ogygia*, as well as that of *H. latidens* and *S. fatalis* is dorsolaterally inclined, whereas in the pantherines this plane is more or less parallel to the sagittal plane (Fig. 8), although in *Pa. onca* the wings are also laterally curved.

**Fig. 9:**
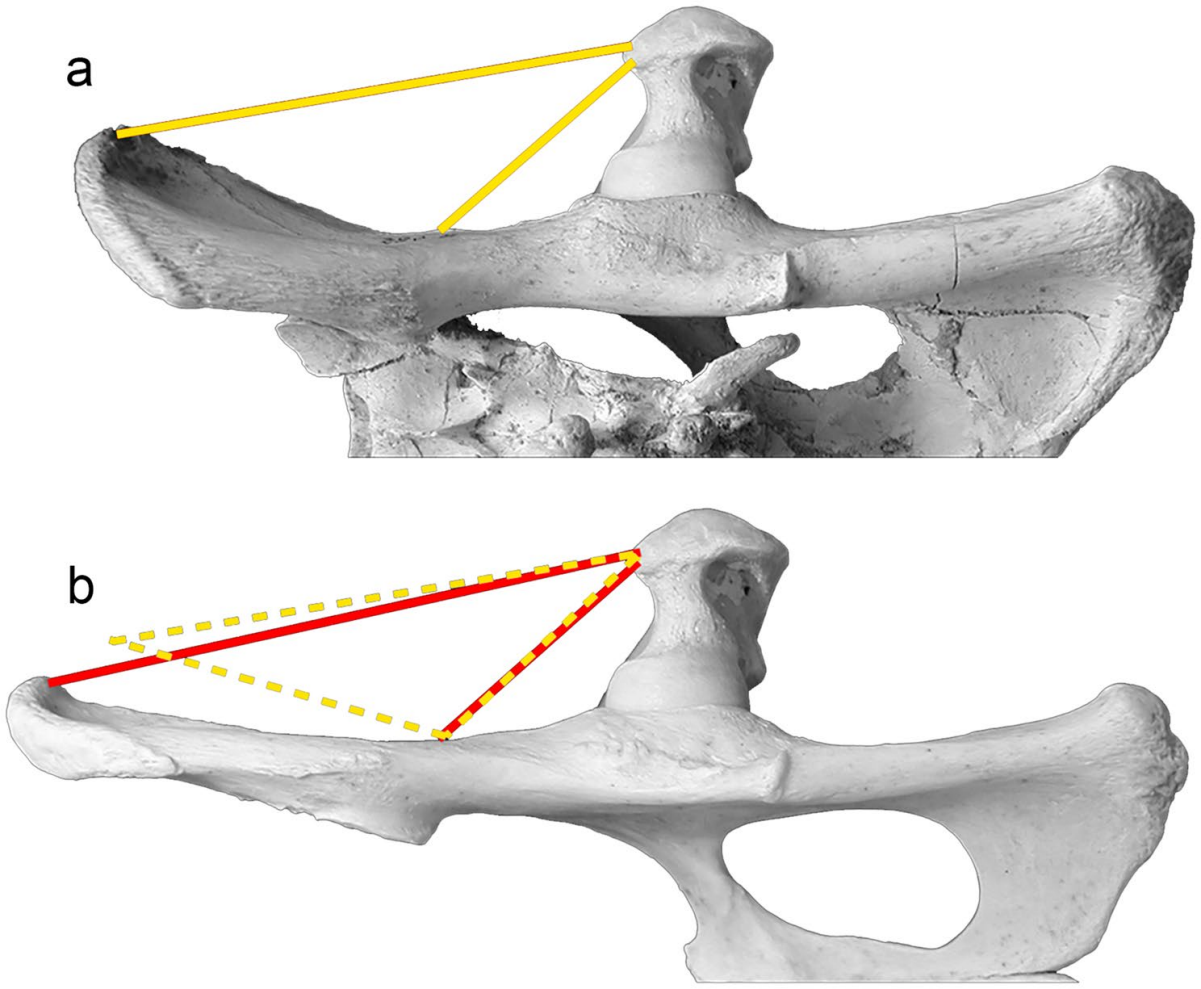
Dorsal view of the pelvis and femur of *Promegantereon* ogygia (**a**) and *Panthera pardus* (**b**) showing the idealized muscular masses of the m. gluteus accessorius and m. gluteus medius, illustrated in yellow in *P. ogygia* and in red in *Pa. pardus*

Owing to this disposition of the iliac wings, in the sabre-toothed felids the attachment surface for the m. gluteus accessorius and m. gluteus medius is clearly observed in dorsal view, whereas in the large felines and pantherines is not visible. The fibres of these muscles originate from the lateral face of the iliac wings of the coxae to the great trochanter of femur [16, 17]. These differences in the orientation of the iliac plane between macairodontines, felines and pantherines would produce two kinds of modifications in these gluteal muscles: (i) the cranial fibres in the sabre-toothed felids would be relatively shorter than those of the felines and pantherines (Fig. 9), and (ii) with the ilium wings being laterally curved, it is very probable that the whole muscular mass of both muscles was relatively thicker in the sabre-toothed felids. Both features basically imply an increase in the strength of contraction of the muscles [30]. Besides this, the iliac wing is relatively shorter in *P. ogygia* than in similar-sized felines and pantherines, which means that the shortening of the longest fibres is even greater. The inclination of the iliac wings plane is lateral and dorsal, and this is probably because the more dorsally disposed fibres decrease their length less than if the inclination were only lateral.

The function of the m. gluteus accessorius is the abduction and inner rotation of the femur, whereas the m. gluteus medius is an extensor of the femur [16]. Both muscles play an important role in hindlimb locomotion, because they contribute to the propulsion of the body during locomotion [16, 31]. The most derived sabre-toothed felids, such as *Smilodon* or *Homotherium*, have a reduced length of the lumbar vertebrae within an overall process of reinforcement and stiffening of the lumbar region [32, 33], which has been related to the necessity for resisting the high muscular stresses produced in the vertebral column when hunting large prey [29, 34]. *Promegantereon ogygia* had a primitive lumbar zone, which is relatively longer than that of derived machairodontines, and with shorter neural process than those of derived machairodontines, as well as those of felines and pantherines; this morphology is highly primitive, and resembles that of viverrids such as *Genetta genetta* [6, 15]. Nevertheless, the shortening of the iliac wings in *P. ogygia* with respect to felines and pantherines means a separation from the primitive model, and represents the beginning of the machairodont pelvic adaptations. Thus, given the importance of the m. gluteus accessorius and m. gluteus medius in the hindlimb locomotion, its reinforcement in *P. ogygia*, as well as the shortening of the iliac wings, could be interpreted as adaptations for generating stronger forces and for supporting higher tensions in that area in comparison to the large felines and pantherines. Stronger muscles and a more robust pelvis support our hypothesis of an adaptation for shortening the initial phase of hunting as the origin of the canine shear-bite [4, 6]. Within this scenario, the larger m. gluteus accessorius and m. gluteus medius, which are capable of generating greater forces, would have provided *P. ogygia* with a biomechanical advantage when controlling and subduing prey in less time, and thus killing it in a few minutes, but also producing an explosive propulsive force during the initial phase of running. Both actions were crucial for the hunting success of *P. ogygia*, considered an ambush predator inhabiting relatively wooded environments [5], which was able to develop a fast and powerful running during short distances, such as seen in extant jaguars or leopards, in order to rapidly catch and immobilize its prey. 

### Sacrum and caudal vertebrae

**Fig. 10:**
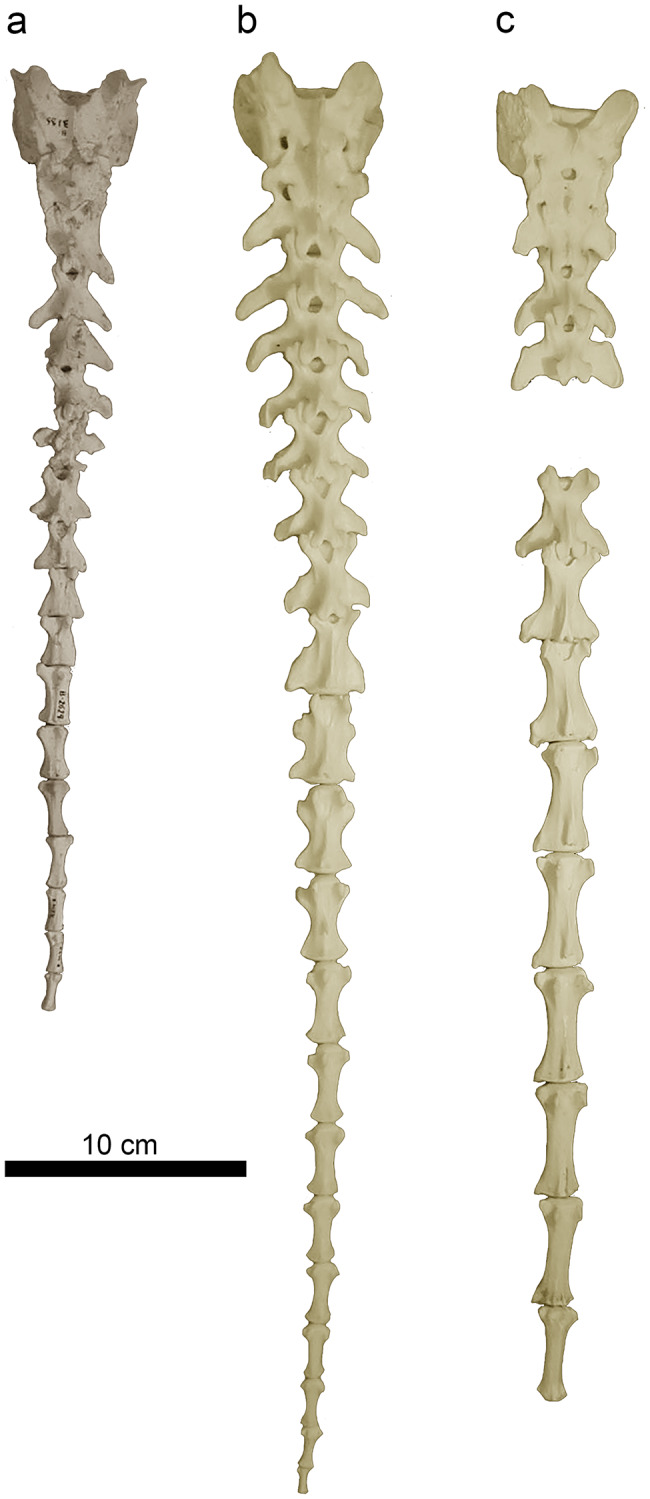
Dorsal view of the sacrum and caudal vertebrae of the compared species of Felidae: **a**, composite of the sacrum B-3185 and several caudal vertebrae of *Promegantereon ogygia*; **b**, MAV-2415, *Panthera onca*; **c**, MAV-2332, *Puma concolor*

The sacrum of *P. ogygia* is relatively slender and elongated in comparison to those of large felines and pantherines, and more similar to that of lynxes. The most interesting feature is the relatively narrow caudal end of the body, as it implies the presence of relatively narrower caudal vertebrae than those of the large felines and pantherines with rectangular sacra, and of a shorter tail (Fig. 10). The presence of an elongated caudal region in the Felidae could be considered a primitive feature, typical of a hypothetic arboreal ancestor of this family, which used its tail to balance when moving along the branches, similarly to other primitive mammals [35–37]. Nevertheless, there are no known sacra of the earliest felid, *Proailurus lemanensis*, or even of other early Feliformia, so the plesiomorphic state of this feature remains unsolved. Besides its function in balance during locomotion, the felid tail has also a relevant role in intraspecific communication, often exhibiting contrasting, very visible spots and stripes, such as those seen in *Pa. pardus*, *Pa. tigris* or *Pa. leo* [38]. Nevertheless, the reduction of the tail observed in sabre-toothed felids would not necessarily imply the loss of that communication function. In fact, a short tail in felids does not have even clear biomechanical implications, as this pattern is observed in slender, fast animals such as the Iberian Lynx (*Lynx pardinus*) a specialist in hunting rabbits; these small mammals, as in many other prey, develop a fast zig-zag running which includes sudden changes of direction when escaping from predators, to avoid capture [39]. In this scenario, it would be expected that *Ly. pardinus* had a long tail, capable of controlling the necessary changes of direction in response to the rabbit’s escape (e.g., similar to the long-tailed cheetah, which uses its tail to help change direction when chasing zigzagging gazelles), but in contrast, this feline has a very short tail. Besides this, several species of sabre-toothed felids show a reduced tail, although their proportions and locomotor adaptations show marked differences. Thus, *S. fatalis*, *S. populator* and *Me. cultridens* were relatively robust animals, with short limbs, whereas *H. latidens* was a much lighter and slender animal, with relatively longer limbs (especially the forelimb, which probably resulted in a sloping back like in the hyaenas). In all these species the tail was as reduced as in the lynxes, even though their hunting strategies were probably very different, with *Smilodon* spp. being considered as ambush predators [13, 27, 29, 40, 41], whereas *H. latidens* has been described as an inhabitant of open landscapes, adapted to hunt fast ungulates after a long pursuit [29, 32, 33, 42, 43]. In both cases, the tail reduction does not seem to have affected their hunting success, so the length of the tail cannot be linked to a specific ecological or functional strategy. In this respect, *P. ogygia* had a relatively reduced tail when compared to those of extant large felines and pantherines, but longer than those of other machairodontines such as *Me. cultridens*, *S. fatalis* and *H. latidens*, which developed very short tails, with fewer and shorter caudal vertebrae than those of large felines and pantherines [20].

### Femur

**Fig. 11:**
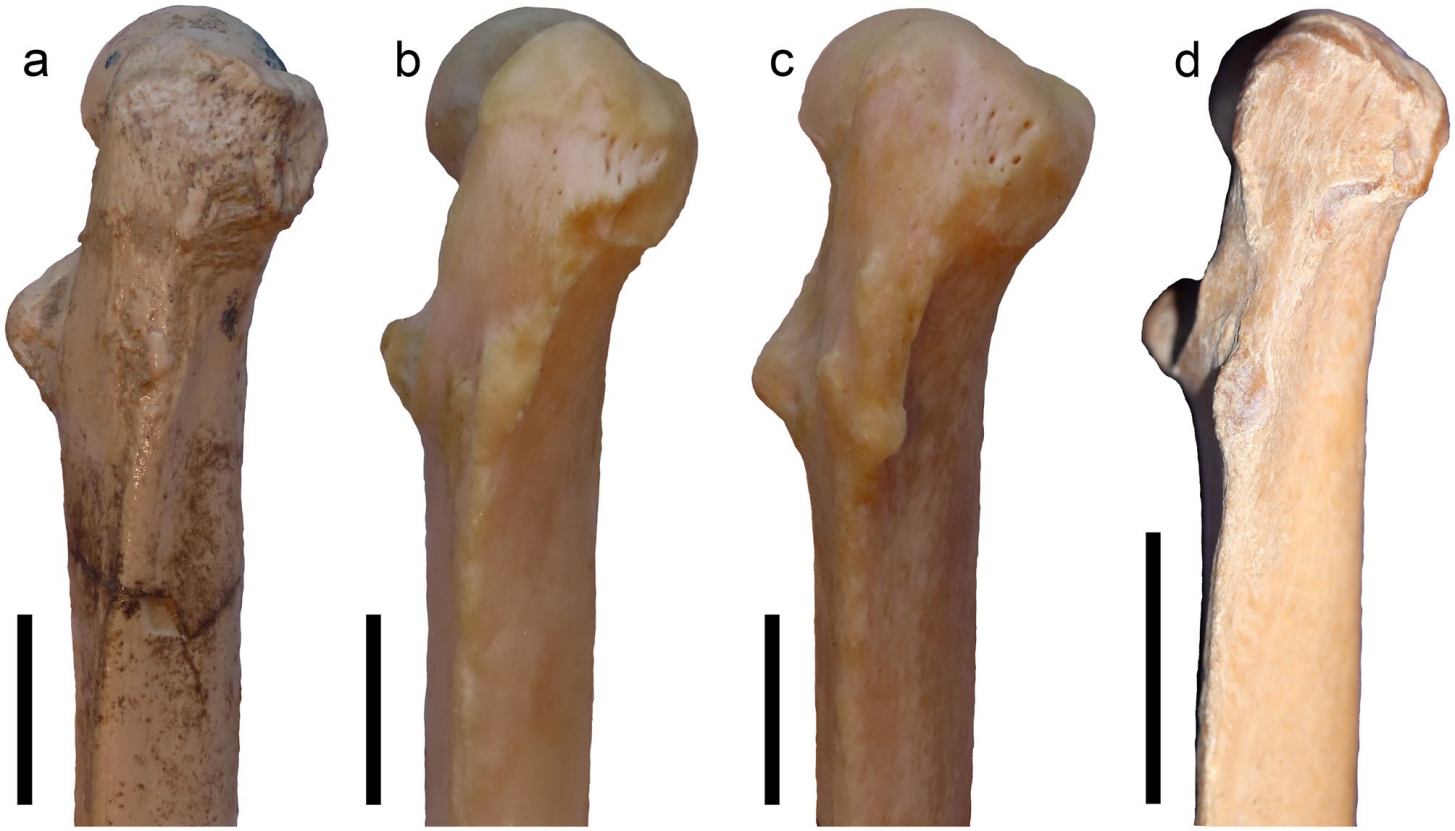
Lateral view of right femora of the compared species of Felidae: **a**, B-2561, *Promegantereon ogygia*; **b**, MAV-2240, *Panthera pardus*; **c**, MAV-2415, *Panthera onca*; **d**, Sg-3529, *Proailurus lemanensis* (showed reversed for better comparison). Each bar represents 2 cm

The femur of *P. ogygia* shows a slightly mediolaterally narrower greater trochanter in caudal view than all the compared species (Fig. 11). This suggests a slight reduction in the attachment areas for the m. gluteus medius, m. gluteus profundus and m. piriformis, which are the major extensors and abductors of the leg, with an important role in the propulsion of the body [16]. Moreover, the greater trochanter is even larger in *Pa. onca*, *H. latidens* and *S. fatalis*, in which this structure is craniocaudally longer in caudal view. The less developed greater trochanter of *P. ogygia* is also observed in more primitive felids, such as *Proailurus lemanensis* and *Miopanthera lorteti*, which suggests that relatively reduced extensor and abductor muscles of the leg was probably a primitive character for the Felidae.

**Fig. 12:**
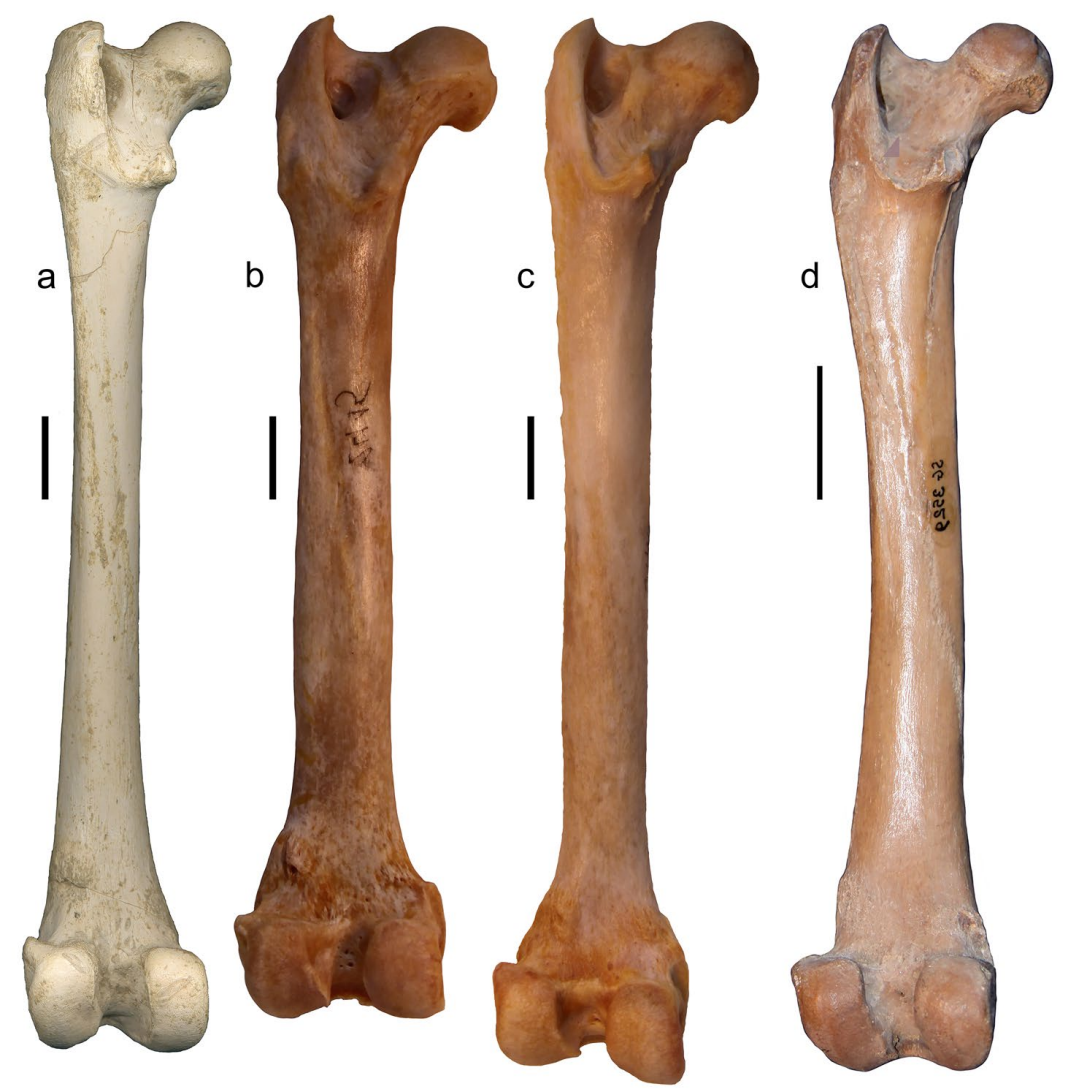
Caudal view of right femora of the compared species of Felidae: **a**, B-2561, *Promegantereon ogygia*; **b**, MAV-2415, *Panthera onca* (shown reversed for better comparison); **c**, MAV-2240, *Panthera pardus*; **d**, Sg-3529, *Proailurus lemanensis* (shown reversed for better comparison). Each bar represents 2 cm

The less excavated trochanteric fossa observed in *P. ogygia* (Fig. 12) suggests a lesser development of the m. obturator externus, m. obturator internus and mm. gemelli, which are all attached in carnivorans into this groove [16, 21, 44–47]. The m. obturator internus is the largest of the three in many carnivorans, although previous studies on Felidae have shown that this difference is not so marked in some species [48–50]; this muscle is a lateral rotator of the coxofemoral articulation, an action in which it is assisted by the smaller mm. gemelli [16]; on the other hand, the m. obturator externus is also a rotator of the coxofemoral articulation [16, 17]. All of these are relatively short muscles, which produce strong contractions, playing an important role in the stabilization of the hip [50]. The morphology of the trochanteric fossa of *P. ogygia* suggests less developed m. obturator externus, m. obturator internus and mm. gemelli, and thus the hip stabilization would have been less optimized than in extant felines and pantherines of similar size. Once again, this sabre-toothed felid shows the primitive morphology observed in *Pr. lemanensis*, which illustrates a mosaic pattern of evolution of the former.

### Tibia

**Fig. 13:**
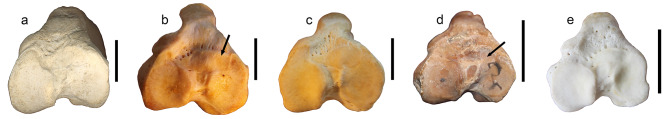
Proximal view of left tibia of the compared species of Felidae: **a**, B-165, *Promegantereon ogygia*; **b**, MAV-2240, *Panthera pardus*; **c**, MNCN-21,566, *Panthera uncia*; **d**, Sg-3516, *Proailurus lemanensis*; **e**, MAV-8169, *Lynx pardinus*. Each bar represents 2 cm. Black arrows indicate the attachment facet for the medial meniscus and its corresponding transverse ligament

One of the most interesting differences between felines, pantherines and machairodontines is observed in the proximal epiphysis of the tibia, and refers to the presence in the two former groups of an elliptical expansion in the cranial margin of the medial condyle, which expands the attachment surface of the medial meniscus and its correspondent transverse ligament (Fig. 13). This accessory facet is also present in other extant felines such as *Felis silvestris*, *Lynx pardinus* and *Acinonyx jubatus*, and in the extinct feline *Miopanthera lorteti*; it is absent in the machairodontines *P. ogygia*, *Me. cultridens*, *H. latidens* and *S. fatalis*. Interestingly, in *Proailurus lemanensis* the medial condyle exhibits a small round expansion on its cranial margin, which could be considered as the beginning of the elongated facet seen in felines and pantherines. However, in viverrids, such as *Genetta genetta*, this accessory facet is absent. The presence or absence of this accessory facet, although allowing an anatomical differentiation between felines, pantherines and machairodontines, does not have clear functional implications. The facet seems to be the attachment area for the cranial cruciate ligament, which has an important role in the biomechanics of the knee: this ligament tenses during knee flexion, restricting the sliding of the tibial plateau in a cranial direction [16].

**Fig. 14:**
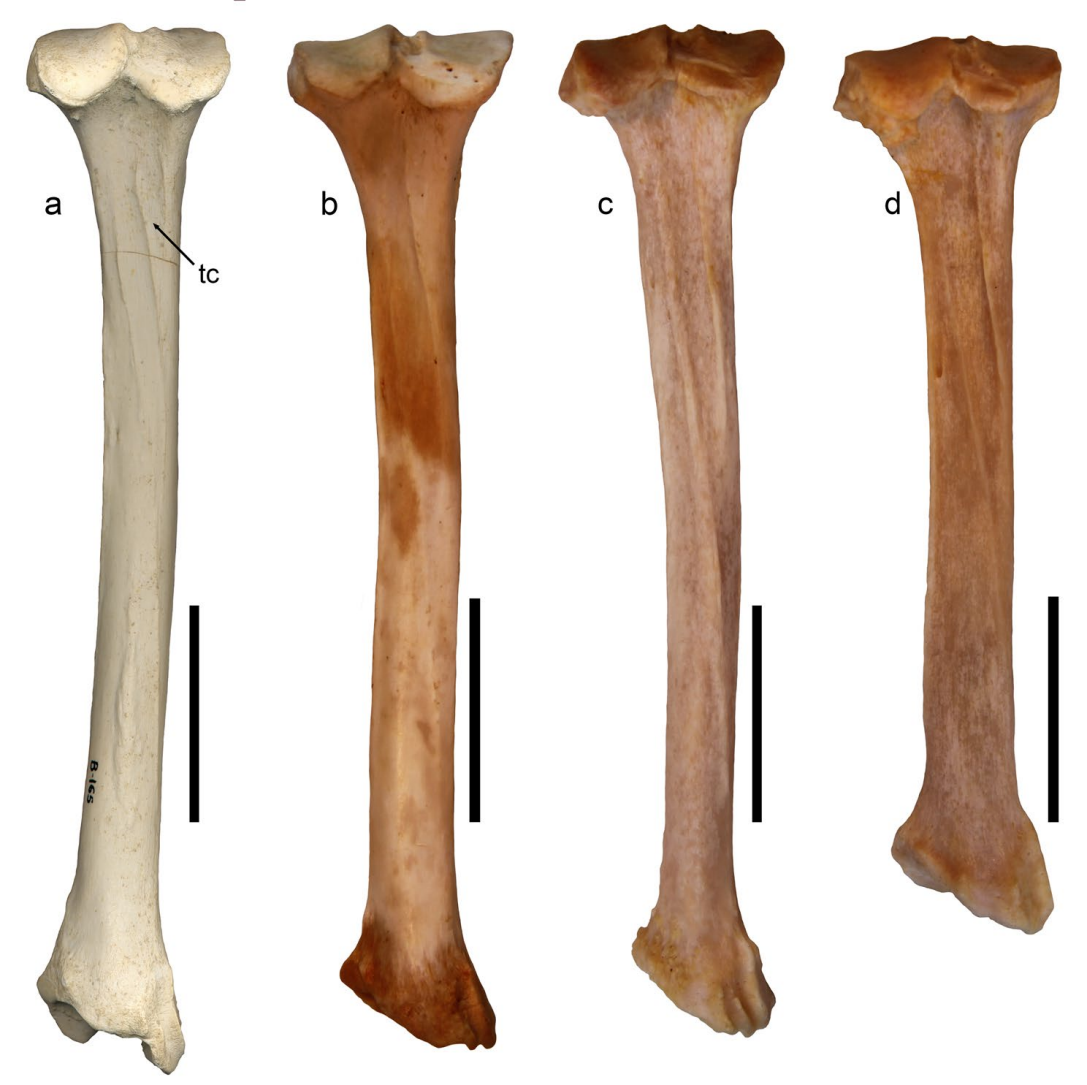
Caudal view of left tibia of the compared species of Felidae: **a**, B-165, *Promegantereon ogygia*; **b**, MNCN-21,566, *Panthera uncia*; **c**, MAV-2240, *Panthera pardus*; **d**, MAV-2415, *Panthera onca*. Each bar represents 5 cm. Abbreviations: **tc**, attachment area of the m. tibialis caudalis

On the caudal face of the diaphysis, the most striking difference between the compared felines and pantherines, and *P. ogygia* is the smaller area (in relation to the total length of the tibia) for the attachment of the m. tibialis caudalis in this later taxon (Fig. 14). This muscle is an extensor of the foot [16], but it is relatively smaller when compared to other muscles of the caudal surface of the tibia: for example, in *Pa. trigris*, this muscle is only the 2.87% of the total mass of the muscles attaching on this area, whereas the m. flexor digitorum lateralis is much larger, reaching the 15.25% [21]. Given this, the relatively smaller size of the m. tibialis caudalis in *P. ogygia* would imply a weaker extension of the feet, which is surprising, as the morphology of the calcaneus suggests a strong flexion of the toes in this species (see below). In this regard, when we assess the relative development of this muscle it is important to take into account the proportion between its narrow, elongated attachment surface and the total length of the tibia; in *P. ogygia*, the slender, elongated proportions of the tibia further remark the proportionally small size of the m. tibialis caudalis in this primitive sabre-toothed felid.

### Talus

**Fig. 15:**
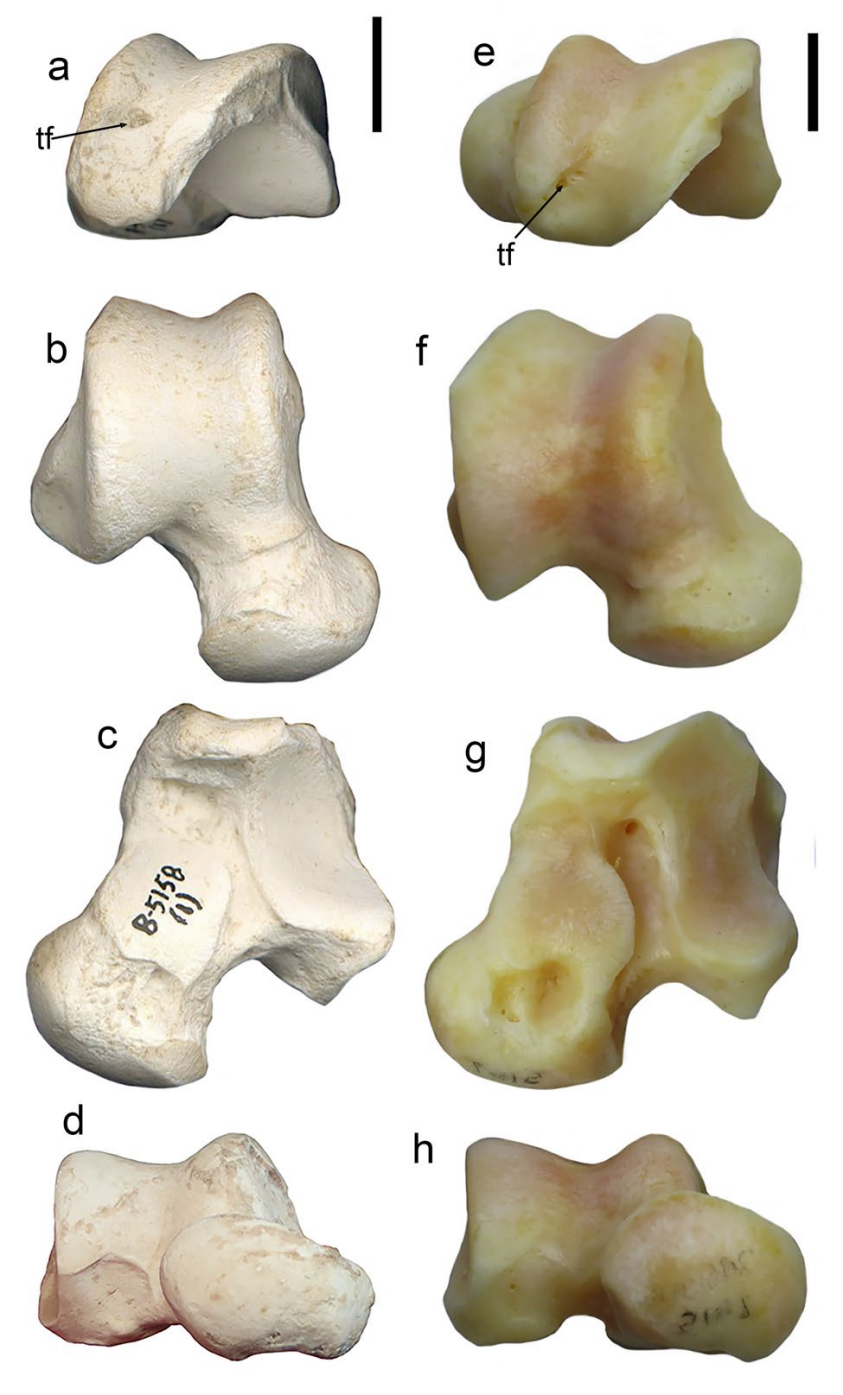
Right tali of the compared Felidae: **a**–**d**, B-5158 (1), *Promegantereon ogygia*, and **e**–**h**, MAV-2415, *Panthera onca*, in **a**, **e**, proximal view; **b**, **f**, dorsal view; **c**, **g**, plantar view; **d**, **h**, distal view. Each bar represents 1 cm. Abbreviations: **tf**, talar foramen

The talar foramen is located more dorsally in *P. ogygia* than in the extant compared felines and pantherines, and the proximal margin of the trochlea is less plantarly elongated (Fig. 15a, e). This is interesting, as the articulation surface for the tibia is thus relatively reduced in the talus of *P. ogygia* compared to those of similarly-sized extant felines and pantherines; this would suggest that the latter would have wider ranges of flexion and extension movement in the ankle due to a longer articular surface between the tibia and talus. In fact, the degree of development of the talar foramen has been related to the multi-planar motion and extension range of the tibial articular surface over the talus trochlea [51] and also with the transition from arboreal to cursorial (those showing adaptations for running) forms [14]. In the viverrid *Genetta genetta*, and in the small feline *Leopardus wiedii*, both very capable climbers [38, 52, 53], the talar foramen is located as in *P. ogygia*; interestingly, this condition is also observed in the earliest known felid, *Proailurus lemanensis*, which would thus illustrate the primitive state of this character for Felidae. Thus, a more plantarly located talar foramen would constitute a derived condition, and would suggests a higher range of extension/flexion movements in comparison to those of *G. genetta*, *L. wiedii*, *Pr. lemanensis* and *P. ogygia*. Nevertheless, it is not clear when this morphology was acquired by the felines, as the Middle Miocene *Miopanthera lorteti*, besides other primitive features for Felidae [54], also shows a more dorsally located talar foramen than its extant relatives. This is interesting, as climbing adaptations would benefit from the development of a trochlear surface that allows wide extension and flexion movements of the ankle, but the taxa with known or inferred climbing abilities show a lower range. In any case, *P. ogygia* would be exhibiting the primitive felid morphology, but this does not exclude a possible ability for climbing, given that it was similar in size to a leopard [5]. In *Me. cultridens* we find the same morphology as in *P. ogygia*, whereas in *S. fatalis*, the last of the Smilodontini, the talar facet can be huge in some specimens [20], which would have limited ankle movement range, but this animal had robust proportions and had a body mass in excess of the lions and tigers, which suggests that it was probably fully terrestrial. In any case, although increasing the ankle flexion and extension range in extant large felines and pantherines would have an obvious advantage in activities such as running, jumping or climbing, the fact that sabre-toothed felids retained the primitive condition for Felidae, could imply subtle differences in ankle biomechanics.

There are other minor differences between the talus of *P. ogygia* and those of the compared felines and pantherines, such as the relatively longer neck, mostly when compared to *Pa. onca* (Fig. 15b, f), the swallower sulcus tarsi (Fig. 15c, g), or the elliptical articular head (Fig. 15d, h). 

### Calcaneus

**Fig. 16:**
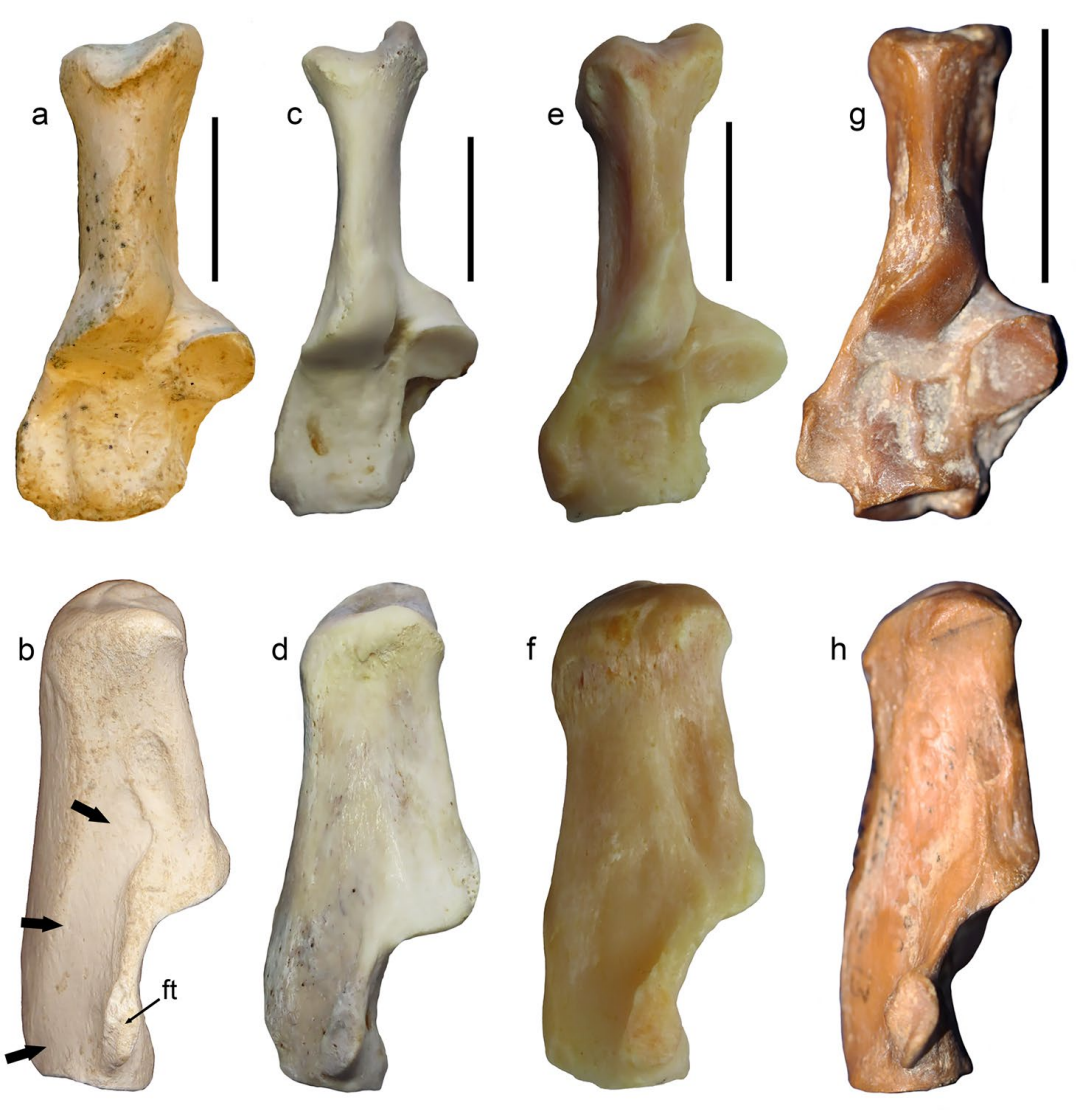
Right calcanei in dorsal (upper) and lateral (lower) of the compared Felidae: **a**,** b**, B/S-341, *Promegantereon ogygia* (shown reversed for better comparison); **c**,** d**, MAV-2332, *Puma concolor*; **e**,** f**, MAV-2415, *Panthera onca*; **g**, **h**, Sg-3517, *Proailurus lemanensis* (shown reversed for better comparison). Each bar represents 2 cm. Black arrows indicate the attachment area for the m. quadratus plantae. Abbreviations: **ft**, fibular tubercle

The fibular tubercle shows differences between *P. ogygia* and the extant large felines and pantherines, with that of the former being more laterally projected (Fig. 16). This structure separates the tendon of the m. fibularis longus from that of the m. fibularis brevis [55]. The more laterally projected fibular tubercle of *P. ogygia* could be a way to increase the space for accommodating the muscular mass of the m. quadratus plantae, which attaches to the lateral surface of the calcaneus, just plantarly to the fibular tubercle [21]. In fact, in *P. ogygia* the attachment area for this muscle is comparatively larger and more excavated than those of the compared felines and pantherines (Fig. 16). Carnivorans in general have reduced this muscle in relation to other groups of mammals, such as primates, in which this muscle is large and divided into two branches, one lateral and one medial [16]. In carnivorans this muscle has only one branch, the lateral one, which is developed from the lateral surface of the calcaneus to the lateral margin of the tendon of the m. flexor digitorum longus, which originates on the proximal epiphysis of the fibula, attaching, by mean of four tendons on the distal phalanxes of the toes [16, 17]. The function of the m. quadratus plantae is to flex the toes [16] and thus its relative size and development have been related to the ability to flex the toe without the contraction of the m. flexor digitorum longus [29]. In those felid species occupying open spaces such as *Pa. leo* and *A. jubatus*, the m. quadratus plantae is very reduced, it being restricted to a thin muscular sheet attached to the distal margin of the lateral face of the calcaneus [56]. This can be related to the development of cursorial abilities in these felids, which also include the lightening of the distal portion of the limbs. In the most arboreal of the felids, *Leopardus wiedii*, as well as in the viverrid *G. genetta*, this muscle is much larger, and the calcaneus shows an elongated, very well-marked attachment area on its lateral surface. The calcaneus of *P. ogygia* shows a similar attachment area for the m. quadratus plantae as that seen in *L. wiedii*, whereas in the extant forest-dwelling pantherines, such as *Pa. pardus* or *Pa. onca*, the attachment area is much less proximally elongated, which suggests a relatively smaller m. quadratus plantae. The presence of a large m. quadratus plantae in *L. wiedii* can be related to the arboreal habits of this species [38, 52], because this muscle would increase the strength of the toes when climbing. *Promegantereon ogygia* shows a similar development of the attachment area in the calcaneus, so the muscle should have been also important in the locomotion of this primitive sabre-toothed felid, both related to climbing abilities and to the development of a powerful propulsion in the initial phase of ambushing.

## Conclusions

**Fig. 17:**
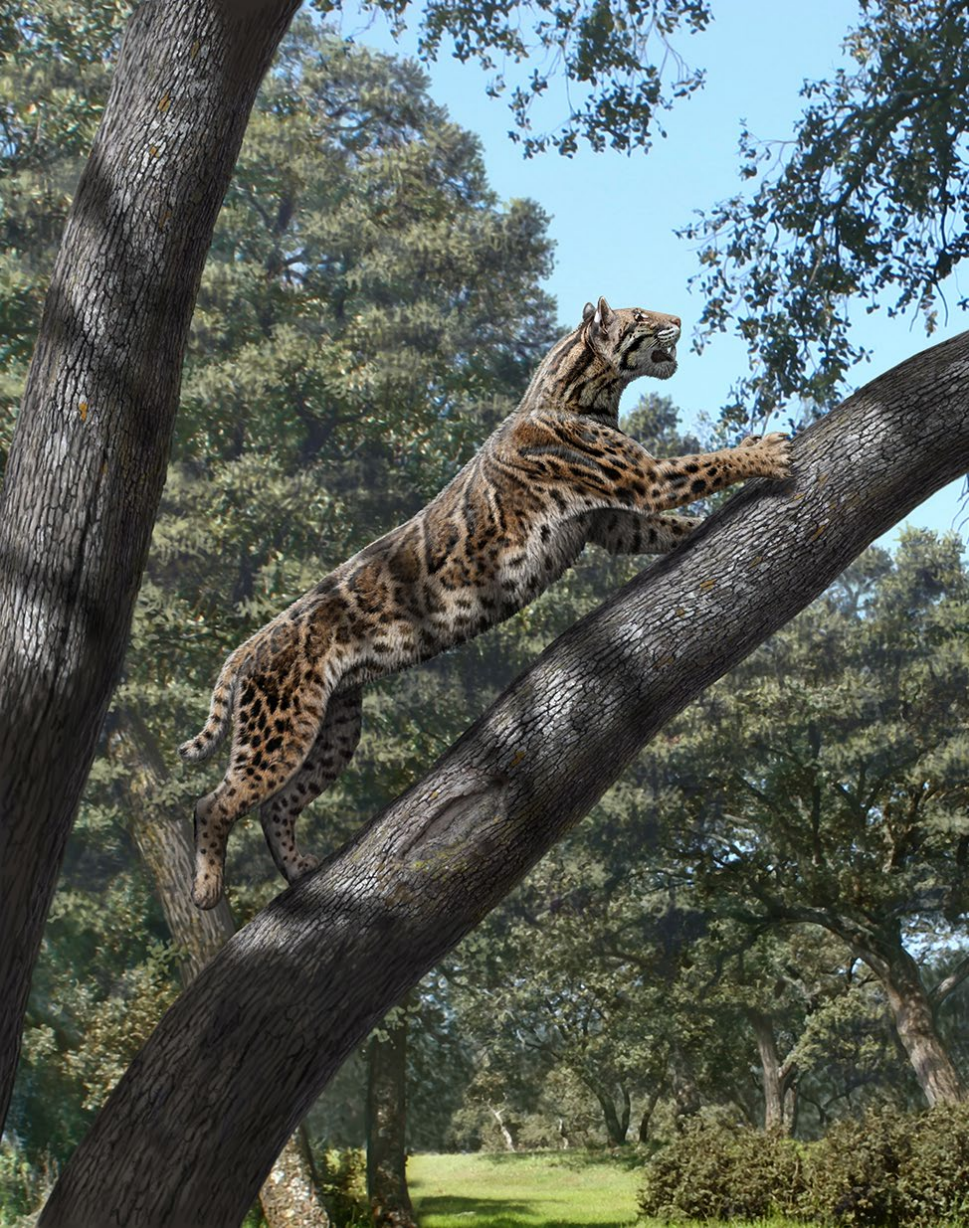
Reconstruction of the life appearance of *Promegantereon ogygia* (artwork by M. Antón)

Our functional analysis of the hindlimb of *P. ogygia* shows that this Late Miocene early machairodontine exhibited several primitive features in its pelvic limb, but also a derived coxae, showing adaptations for developing strong propulsive forces and also to resist the struggles of captured prey, both actions probably linked to the sabre-toothed hunting and killing method. *Promegantereon ogygia* has been described as an ambush predator that inhabited relatively wooded environments, such as those inferred for the surroundings of the Batallones-1 natural trap [5]. In that scenario, this predator developed powerful forelimbs, well suited for grasping and subduing prey during hunting [6], but also, as shown by our study, a specialised coxae, built for developing a rapid and explosive initial phase of prey pursuit, which was very powerful but lasted only a short period of time, such as seen in extant jaguars or leopards do [57–61]. A specialised coxae would have helped not only during the pursuit of prey, but also during its capture and subduing, when *P. ogygia*, used the powerful muscles of the forelimb and coxae to immobilize its prey on the ground, avoiding its struggling movements when trying to escape and allowing to deliver with safety the killing bite [4, 5, 27, 29, 62]. Besides this, it is remarkable that other elements of the pelvic limb do not show these adaptations and, for example, the morphology of the proximal epiphysis of femur, suggests relatively less developed m. obturator externus, m. obturator internus and mm. gemelli than those of extant similarly-sized felines and pantherines, implying a less optimized hip stabilization. Another example is the large excavated area for the attachment of the m. quadratus plantae on the lateral face of the calcaneus of *P. ogygia*, which suggests a great capacity for flexion of the toes; this feature, besides being a primitive condition for Felidae, would increase the climbing abilities of this sabre-toothed felid (Fig. 17).

In summary, our study also shows a marked difference existing in the evolutionary stages of the forelimb and hindlimb of *P. ogygia*: whereas the former already displayed a set of derived features related to an increase in the grasping and flexion force of the hand, elbow and shoulder [6], the hindlimb was basically primitive, with several characters showing a closer morphology to that of the early felid *Proailurus lemanensis*, and far from the pelvic limb anatomy seen in other more derived sabre-toothed felids. In fact, the only derived feature of the hindlimb of *P. ogygia* is the coxae, which shows a morphology adapted to generate strong propulsive forces during locomotion, as well as to control the lateral movements of the back, which is related to the sabre-toothed felids hunting method. This highlights the pattern of mosaic evolution seen in this early form of Late Miocene Machairodontinae, and emphasizes the importance of studying the skeletal anatomy of more primitive forms, such as the Middle Miocene genus *Pseudaelurus*, which will be the focus of our future research. 

## Data Availability

The fossils of *Promegantereon ogygia* analysed during the current study are available in the collections of the Museo Nacional de Ciencias Naturales-CSIC (Madrid, Spain) and Museo Arqueológico y Paleontológico de la Comunidad de Madrid (Alcalá de Henares, Spain), with the following catalogue numbers: sacra: B-2684, B-199 (1), and B-4651; coxae: B-767, B-2684, B/S-540, B/S-552, B-4326, B-3938, B-4374, B- 2045, B-466, B-199 (1), and B-199 (2); femora: B-765, B-766, B-768, B-2033, B-2681, B-2682, B/S-557, B-3885, B-3123, B-447, B-2561, B-625, B/S-681, B-4709, B-1783, B-4456, and B-1826; tibiae: BAT-1’05 F6-42, BAT-1’24 92, B-4380 (1), B-769, B-155, B-4645, B-784, B-2683, B/S-564, B/S-548, B/S-545, B/S-556, B-344, B-3155, B-471, B-811 (11), B-4940, B-788, B- 711, B-3330, B-744 (12), B-3379, B-2703 (3), B-2588, B-1348, B-1336, B- 5415, B-1497, B-4650, and B-5140 (2); calcanei: B-4818c, B-5158 (2), B-791 (9), B-1172 (2), B-749 (2), B-159, B-235, B- 153, B-119, B-3467, B-1065, B-4399, B-81 (1), B-2678 (1), B-4380 (2), B- 4512 (1), B/S-341, B-3880, B-6049, B-2684, B/S-349, B/S-356, B/S-337, B/S-351, B/S-357, B/S-339, B/S-374, B/S-345, B/S-340, B/S-346, B-3249 (1), B-3343, and B-3963; tali: BAT-1’02 F4-53, B-4818d, B-3881, B-177, B-4398 (1), B-817 (1), B-2241 (1), B-5158 (1), B-1172 (3), B-791 (9), B-749 (1), B-5128, B-528, B-5289, B-1993, B-547, B-4204, B- 1854, B-4419, B-147, B-120 (4), B-81 (2), B-4380 (3), B-2678 (2), B-4512 (2), B-792 (30), B-6050, B-2684, B/S-373, B/S-334, B/S-344, B/S-361, B/S- 330, B/S-412, B-3421, B-3249 (2), B-3633, and B-4816.

## Abbreviations

lig.: ligament
m.: muscle
mm.: muscles
MNCN: Museo Nacional de Ciencias Naturales-CSIC
MAV: Museo Anatómico de la Universidad de Valladolid
n: number of elements
SD: Standard deviation
tc: attachment area of the m. tibialis caudalis
ft: fibular tubercle
tc: attachment area of the m. tibialis caudalis
tf: talar foramen
